# A review on the role of deep eutectic solvents in mango (*Mangifera indica*) extraction

**DOI:** 10.1039/d5ra00097a

**Published:** 2025-02-10

**Authors:** Ahmad Mukhlis Abdul Rahman, Amirul Ridzuan Abu Bakar, Ang Qian Yee, Mohd Asraf Mohd Zainudin, Nik Muhammad Azhar Nik Daud, Ahmad Anas Nagoor Gunny, Mohd Sharizan Md Sarip, Ryan Vitthaya Peron, Nurul Husna Khairuddin

**Affiliations:** a Faculty of Chemical Engineering & Technology, Universiti Malaysia Perlis Jejawi Perlis 02600 Malaysia amirulridzuan@unimap.edu.my; b M. Kandiah Faculty of Medicine and Health Sciences, University Tunku Abdul Rahman Bandar Sungai Long Kajang Selangor 43000 Malaysia

## Abstract

The present review attempts to evaluate the applicability of deep eutectic solvents (DES) as a green technique for the extraction of phytochemicals from *Mangifera indica* L. and their therapeutic potential. Mango has been reported to show numerous therapeutic activities, which are attributed to its abundant source of bioactive compounds. Thus, the therapeutic potential of phytochemicals in mangoes is reviewed based on different reported bioactivity tests. The use of DESs is considered a green approach for the extraction of bioactive compounds from natural sources utilizing two or more components and a safe alternative for application in the nutritional, pharmaceutical and other sectors. The trends in the extraction of phytochemicals from mango using different DES components and different extraction parameters of the optimum protocol are reviewed. Hence, DESs are considered potential solvents with selective and efficient properties for extracting bioactive ingredients from mango. However, there are several knowledge gaps that need to be assessed for DES-based bioactive compound extraction from mango such as information on the local and specific varieties of mangoes, standardization of the extraction protocols and use of other parts of the mango plant as alternatives to its peel as bioactive sources. Accordingly, the extraction of bioactive compounds from mango using DESs will provide useful information for subsequent agricultural, pharmaceutical and nutraceutical applications in the future.

## Abstract 2

The present review attempts to evaluate the applicability of deep eutectic solvents (DES) as a green technique for the extraction of phytochemicals from *Mangifera indica* L. and their therapeutic potential.

## Graphical abstract

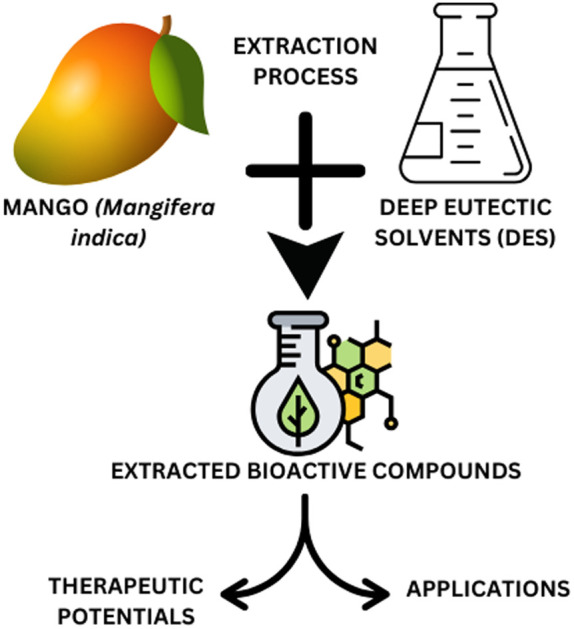

## 1. Introduction

Phytochemicals are naturally occurring compounds in plants that can be divided into primary and secondary metabolites. Primary metabolites such as nucleic acids, carbohydrates, fatty acids and proteins are required for the normal growth and development of plants.^1^ Alternatively, secondary metabolites are compounds produced by plants for their protection and to enhance their ability to survive in harsh environments. Phytochemicals including secondary metabolites can be derived from various sources such as medicinal plants,^2,3^ vegetables,^4,5^ fruits,^6–8^ and agricultural by-products.^9–11^ Secondary metabolites are important plant constituents with effective therapeutic potential, especially exhibiting antioxidant and anthelmintic activities.^12–14^ Phytochemicals can be found in different parts of a target plant, such as stems, roots, seeds, fruits, leaves and flowers. The extraction of phytochemicals using different plant parts usually produces different extraction yields and types of phytochemical compounds.^15,16^

To date, the recovery of bioactive compound extracts from several natural sources is highly associated with the conventional extraction process using conventional extraction techniques such as percolation, maceration, digestion, decoction and Soxhlet extraction in combination with traditional organic solvents.^1,17–19^ Examples of traditional organic solvents are acetone, ethyl acetate, methanol and ethanol.^17,20–23^ However, conventional extraction techniques are usually based on highly pollutant, non-renewable organic solvents and often related to increased energy consumption, toxicity and volatility.^24–26^ Therefore, there is an urgent need to apply green extraction approaches, which are considered safer and more environmentally friendly as well as reduce water, solvent and energy consumption.^26,27^

Ionic liquids (ILs) are the first multimolecular generation of solvents being used as green solvents for the extraction of bioactive compounds. The advantages of IL solvents include their wide range of miscibility and solubility, very low vapor pressure, non-flammability and good thermal properties and recyclability.^20,27,28^ However, ILs do possess several disadvantages such as high production cost, high toxicity, poor water stability, difficult purification and low biodegradability.^27–30^ Thus, over the past few years, the scientific community has been committed to applying deep eutectic solvents (DESs) for the recovery of bioactive compounds. DESs have been developed as analogous to ILs with similar physicochemical properties. The greener and environmentally friendly DESs have been reported to have additional advantages compared to ILs including biodegradable properties, chemical inertness with water, lower toxicity and lower cost.^20,31^

However, they differ from ILs in several aspects such as the source of their starting materials and chemical formation process. The formation of DESs is a result from the chemical reaction between a halide salt or another hydrogen bond acceptor (HBA) and a hydrogen bond donor (HBD). Natural deep eutectic solvents (NADESs), new derivatives of DESs, have been developed from the combination of primary metabolites and bio-renewable materials such as sugar, alcohols, organic acids and amino acids.^31,32^ Accordingly, NADESs may be very useful for solubilizing, storing or transporting non-water-soluble metabolites in living cells and organisms. Several factors that affect the extraction yield of bioactive compounds from different parts of mango by DESs such as the combination of materials for the preparation of DESs and NADES, application of innovative extraction techniques and selected critical extraction parameters are reviewed in this article.

Mango (*Mangifera indica*) is widely consumed worldwide and is rich in bioactive compounds such as polyphenols, carotenoids, vitamins and minerals. It has been used for medicinal purposes for centuries and has potential in various therapeutic effects such as antioxidant,^33^ anti-inflammatory,^34^ anti-cancer^35^ and anti-diabetic^36^ activities. Several different parts of mango have been used for the extraction of bioactive compounds. The main source of mango that has been used for the extraction of bioactive compounds by DES is the mango peel,^37–39^ while other parts of the mango plant have not been widely exploited such as its leaves, kernels, and seeds, which will be useful targets for DES extraction to gain new knowledge on the additional and complementary data of bioactive compounds. This review article also describes the therapeutic potential of bioactive compounds extracted from different sources of mango samples.

## 2. Background of DESs

### 2.1 Historical development of DES

DESs are environmentally friendly solvents similar to ionic liquids (ILs) and are often compared to ILs because they share some common characteristics, such as high thermal stability, low volatility and adjustable polarity. This unique class of solvents gained attention following the discovery by Abbott and colleagues.^40^ These researchers found that when certain substances capable of forming hydrogen bonds, known as hydrogen bond donors (HBDs), are mixed with others that can accept these bonds, referred to as hydrogen bond acceptors (HBAs), in specific proportions, the melting point of the mixture decreases significantly.

This surprising behaviour was first noticed when choline chloride powder (typically melts at a high temperature of about 302 °C) was combined with crystalline urea (with a melting point of around 133 °C) in a 1 : 2 ratio. Remarkably, this mixture turned into a liquid at room temperature, with a low melting point of its eutectic composition of about 12 °C.^40^ Abbott and team named this unique liquid a “deep” eutectic mixture (DEM). DEMs, due to their ability to melt at much lower temperatures, have opened up new possibilities for creating innovative solutes and electrolytes for various chemical applications.^40–48^ Scientists often refer to these liquids as DES when discussing their roles as solvents in specific applications.

Ionic liquids (ILs), which are composed of anions and cations and have a melting point below 100 °C, have captured the attention of organic chemists looking for environmentally friendly and sustainable methods since their discovery in 1914. At that time, no one could have predicted that ILs would become a prominent topic in chemistry a century later. Today, some key perspectives differ from the original concepts as our understanding of ILs has deepened. For example, ILs were initially thought to be non-volatile, non-flammable and stable in air and water, making them appear as green solvents. However, it is now widely recognized that many ILs are indeed volatile, flammable, unstable, and sometimes toxic.^49^

This complexity arises from the vast combinations of cations and anions that meet the definition of ILs, leading to a wide range of behaviours.^49^ In contrast, DESs have several advantages over ILs. Although ILs can be expensive, harmful to the environment and potentially toxic, DESs are typically affordable,^50,51^ environmentally friendly,^52,53^ and safe to use.^53–55^ For instance, choline, a common component in DESs, is found in vitamin B and widely produced for livestock nutrition, making it readily available and harmless.^56,57^ In contrast, urea, a typical component in DESs, is often used in fertilizers but does not share the same eco-friendly characteristics.^58^

### 2.2 Principles and classification of DESs

Deep eutectic solvents (DESs) consist of multiple compounds, with a hydrogen bond donor (HBD) and a hydrogen bond acceptor (HBA), exhibit a considerably lower eutectic point than an ideal liquid mixture and remain in a liquid state at ambient temperature.^59–61^ The physiochemical properties of DESs, encompassing attributes such as their melting point, density, conductivity and viscosity, undergo variations depending on the specific structures of these solvents. The melting point of DESs is primarily determined by the proportion of hydrogen bond donor present in them, resulting in a significantly lower melting point compared to their individual constituents.^29,62^

An increased concentration of hydrogen bond interaction with anionic groups leads to diminished interactions with cationic groups. This shift in interaction, characterized by a reduced lattice energy, contributes to the reduction of the melting point.^63^ The general formula for deep eutectic solvents can be expressed as Cat^+^X^−^*z*Y. The cation, denoted as Cat^+^, can be an ammonium, phosphonium or sulfonium ion, while X^−^ represents a Lewis base, often a halide anion. The variable Y signifies a Lewis or Brønsted acid, and the value of *z* corresponds to the number of Y molecules interacting with the anion X^−^.^26,29^ Most DESs can be grouped into four main types, as discussed in the next subtopic.

#### 2.2.1. Common types of DESs

Type I DESs combines a quaternary ammonium salt with a metal chloride, type II pairs a quaternary ammonium salt with a water-containing metal chloride, type III involves a quaternary ammonium salt and a substance providing hydrogen bonds (HBD) and type IV is formed when a water-containing metal chloride mixes with an HBD.^64^ Type V represents an emerging category of eutectic mixtures, consisting solely of non-ionic molecules of hydrogen bond acceptors (HBAs) and hydrogen bond donors (HBDs), as exemplified by the thymol–menthol deep eutectic mixture.^65^ Although deep eutectic solvents (DESs) share some physical properties with ionic liquids, they differ in terms of their chemical structures. The non-ionic variation of DESs, type V, holds significant importance given that it significantly broadens their properties and potential applications. Additional mixtures that do not fit neatly into these categories, such as a combination of specific Brønsted–Lowry acids and bases, also exhibit deep eutectic behaviour, further broadening the scope of DES exploration.

Type I DESs include well-studied combinations such as chloroaluminate/imidazolium. They are also comprised of less common ionic liquids, such as those made by mixing imidazolium salts with different metal halides, including FeCl_2_.^66^ Additionally, the research by Scheffler and Thomson involved type I eutectics, including EMIC combined with various metal halides, including AgCl.^67^ However, the availability of nonhydrated metal halides suitable for type I DESs is limited due to their higher melting points. Thus, improving deep eutectic solvents involves mixing choline chloride with hydrated metal-halides, resulting in type II DESs. These hydrated metal salts are cost-effective and exhibit resistance to air and moisture, making them suitable for large-scale industrial use.^68^ Type III DESs refer to a category of eutectic mixture where the hydrogen bond donor (HBD) typically consists of an organic molecular component such as amides, carboxylic acids, amino acids, sugars and polyols.

Type III DESs, resulting from the combination of choline chloride with hydrogen bond donors such as amides and carboxylic acids, have attracted increasing attention owing to their versatility and favourable properties. Their capability to effectively dissolve a wide range of transition metal compounds, including chlorides and oxides, contributes to their widespread use across various applications.^29,69–71^ Furthermore, they are easy to prepare, have limited reactivity with water, are often environmentally friendly and are cost-effective. The wide range of available hydrogen bond donors makes this category of deep eutectic solvents versatile and adaptable. Regardless of the specific type, DESs are believed to be formed through interactions involving factors such as the entropy of mixing, van der Waals forces, hydrogen bonding and/or ionic bonding.^29,69–71^ However, further investigation is required to gain a comprehensive understanding of how these factors interact and contribute to the properties of DESs.

Some researchers have proposed expanding the classification of deep eutectic solvents (DESs) by introducing two additional categories, namely, therapeutic DESs (THEDES, type VI) and amino acid-based DESs (AADES, type VII).^72^ Additional classification based on function or dominant components has distinct advantages, such as aiding readers and the scientific community in understanding the relevant types of DESs. Nevertheless, some groups of DESs, which are organized based on important functions or dominant components, such as NADES and VODES, have not been explicitly included in this classification. This matter can be resolved through two approaches.

The first approach involves including NADES and VODES as additional categories within the existing classification. However, this approach may lead to substantial overlap, with some DESs potentially falling into multiple distinct categories, making the situation more complex. Accordingly, the second approach can be considered to avoid this confusion. It involves introducing a new set of classifications based on specific functions, applications and the dominant components of DESs. This new classification is meticulously organized according to the primary functions and dominant components of the relevant DESs. Through this approach, NADES, THEDES, VODES and AADES can be systematically categorized. However, given that this article is not focused on the in-depth examination of new classifications, a thorough analysis of this matter can be systematically discussed by authoritative scientific communities in the future. This may give rise to specialized review papers dedicated to this topic.

#### 2.2.2. Number of components and miscibility of DESs

Besides classifying deep eutectic solvents (DESs) based on their specific components, an alternative classification approach considers the number of constituents present in the solvent mixture. This alternative classification system focuses on the simplicity or complexity of the DES composition and plays a crucial role in understanding and selecting the most appropriate DES for different applications. It distinguishes between “binary” and “ternary” DESs based on the number of components present in the solvent mixture, offering valuable insights into their characteristics and potential applications. Binary DESs are a type of DES composed of two primary components, typically an HBD (hydrogen bond donor) and HBA (hydrogen bond acceptor).

The examples of binary DESs include choline chloride/triethylene glycol,^73^ choline chloride/glycerol,^74^ zinc nitrate hexahydrate/glycerol,^75^ tetraethylammonium chloride/ethylene glycol,^76^ and choline chloride/levulinic acid.^77^ Alternatively, ternary DESs are composed of three main components, *i.e.*, an HBD, an HBA and a third component, often a metal salt or another substance. This additional component adds complexity to the DES composition and can result in distinctive properties or improved performance in specific applications. The examples of ternary DESs include dichloroacetic acid/l-menthol/*n*-butanol,^78^ choline chloride/anhydrous oxalic acid/ethylene glycol, choline chloride/anhydrous oxalic acid/glycerol and choline chloride/anhydrous oxalic acid/lactic acid.^79^

Deep eutectic solvents (DESs), which are created by combining hydrogen bond donors and acceptors, represent a promising and innovative category of solvents. Both hydrophilic and hydrophobic binary DESs have a tendency to absorb water, resulting in the formation of ternary mixtures. Consequently, the presence of a minor water content is practically unavoidable under normal ambient conditions.^80^ Hydrophobic deep eutectic solvents (DESs) are a type of custom-designed solvent known for their limited or even absent water miscibility and low vapor pressure. These DESs are typically created by combining hydrophobic hydrogen bond acceptors (HBAs) and hydrogen bond donors (HBDs).

As a result, hydrophobic DESs tend to separate from water, forming distinct layers or phases due to their limited solubility in aqueous environments. Hydrophobic deep eutectic solvents (DESs), such as the combination of tetrabutyl ammonium chloride and decanoic acid, are characterized by their low water miscibility. Even a minimal water content in these DESs can trigger dynamic nanoscale phase segregation, resulting in reduced solvent viscosity and increased fragility. This phenomenon also leads to higher self-diffusion coefficients and conductivity within the solvent, ultimately enhancing the local dynamics and making hydrophobic DESs valuable in various applications.^80^

Alternatively, hydrophilic deep eutectic solvents are made up of components that have a strong affinity for water. These DESs are formulated by combining hydrophilic HBAs and HBDs, resulting in stable solutions that are highly soluble in water. Choline chloride (ChCl) and lactic acid are typically considered hydrophilic components when used in deep eutectic solvents (DESs).^81^ These components have an affinity for water and can readily form hydrophilic DESs. Hydrophilic DESs are valuable in situations where maintaining a water-compatible environment is essential. They can be employed as environmentally friendly solvents for extracting hydrophilic compounds or as suitable media for hydrophilic chemical reactions that require an aqueous environment.

### 2.3 Properties of density and viscosity in DESs

#### 2.3.1. Density

DESs typically exhibit higher densities than water, with values in the range of 1.0 to 1.3 g cm^−3^ at 25 °C for most reported DESs.^82^ However, DESs based on metal salts tend to have even higher densities, falling in the range of 1.3–1.6 g cm^−3^.^72^ Alternatively, hydrophobic deep eutectic solvents often have lower densities than that of water.^72,83^ The density of deep eutectic solvents (DESs) is a critical property influenced by several factors including their molecular organization, packing, and presence of voids or vacancies. Thus, the density of DESs can vary significantly based on the combinations of hydrogen bond acceptors (HBA) and hydrogen bond donors (HBD). The hole theory is a useful tool to understand these variations, given that it links the properties of DESs to available holes or voids in the solvent, roughly the size of mobile species.

Changes in the density of DESs also result from the dynamics of molecular interactions and the availability of free volume in the DES. As the temperature of the DES increases, more free space becomes available, allowing molecules to move more rapidly. This increased molecular motion leads to a reduction in the density of the DES. The density of deep eutectic solvents (DESs) is clearly affected by the characteristics of the HBD such as the presence of different halide ions in the HBA.^84–86^ For example, DESs containing the bromide salt of tetraalkylammoniums tend to exhibit higher densities compared to that with chloride salts.^87–89^ Additionally, when considering the alkyl chain length of HBD, ranging from ethyl to butyl, in tetraalkylammonium salts, the density of DESs is the highest when using TEABr/Cl. This is primarily due to the compact structure of the DESs.^89,90^

Another example involves ChCl-based DESs, where a range of deep eutectic solvents (DESs) was created by combining choline chloride (ChCl) with various carboxylic acids having different alkyl chain lengths, including oxalic acid, glycolic acid, malonic acid, glutaric acid and levulinic acid.^91^ Particularly, DESs formulated with glutaric acid and levulinic acid exhibited lower densities compared to those composed of other acids. This decline in density was primarily attributed to the longer C5 alkyl chain found in these compounds, and an increase in alkyl chain length resulted in a higher molar volume, subsequently leading to a decrease in the DES density.^91^ Also, the density of DESs is significantly influenced by the molar ratio of HBA to HBD.^86,92–94^ For example, in TBABr : PEG-based DESs, an increase in the molar ratio of PEG resulted in a slight elevation in density.^88^ DESs with a 1 : 3 HBA to HBD ratio displayed higher densities compared to that with a 1 : 2 ratio. Specifically, the densities of TBABr : PEG 200 at 1 : 2 and 1 : 3 ratios were recorded to be 1.11077 g cm^−3^ and 1.11360 g cm^−3^, respectively.

Temperature affects the density of deep eutectic solvents (DESs), and this relationship is described by the isobaric thermal expansion coefficient, which indicates the available free volume in DESs. This coefficient helps explain how DESs compress.^94^ The isobaric thermal expansion coefficient (*αP*) is calculated based on experimental data. DESs exhibit a linear decrease in density with an increase in temperature. This drop is due to the increased free space between the hydrogen bond acceptor (HBA) and hydrogen bond donor (HBD) components. Although the *αP* values of DESs are lower than that of common solvents, they are similar to that of imidazolium-based ionic liquids (ILs). DESs show less expansion or compression with temperature compared to ILs and organic solvents.^94^ Numerous researchers have extensively investigated the density of DESs at different temperature levels.^95–97^

For example, the density of ChCl-based deep eutectic solvents (DESs) was measured at a constant pressure of 101.3 kPa in the temperature range of 293.15 K to 333.15 K.^98^ These DESs were formulated using ChCl as the hydrogen bond acceptor (HBA) and various hydrogen bond donors (HBDs), including ethylene glycol, levulinic acid and phenol, maintaining a consistent 1 : 2 HBA-to-HBD mole ratio. The results consistently revealed that with an increase in temperature, the density of these DESs decreased due to thermal expansion. Among the examined DESs, the ChCl : levulinic acid-based DES demonstrated remarkable thermal stability, which is primarily attributed to the robust intermolecular interactions between ChCl and levulinic acid. These interactions resulted in higher density compared to the other DESs.^98^

Shah and Mjalli conducted a study to explore the impact of water content on the density of a ChCl : urea deep eutectic solvent (DES) at two different temperatures.^99^ To gain insights into the interactions within pure DESs and their aqueous solutions, they used molecular dynamics simulations. The findings revealed significant insights into the behaviour of the DESs. Notably, the presence of urea and Cl anion interactions affected the melting point of the DES. Interestingly, the Cl anions exhibited higher levels of hydration compared to urea and choline cations. These interactions had a direct influence on the melting point, given that they interfered with the interactions between the urea molecules and choline cations.

The impact of water on density was divided into three distinct phases. As the content of water increased, the number of hydrogen bonds within the DES decreased. At higher concentrations of water, the anion of the hydrogen bond donor exhibited increased hydration compared to its cation.^70,94^ These findings were consistent with the observations for ChCl : urea under high-pressure and temperature conditions, ranging from 298.15 to 323.15 K. Yadav and Pandey conducted a study on aqueous solutions of this deep eutectic solvent in the temperature range of 298.15 K to 363.15 K. They formulated a quadratic equation to demonstrate the decrease in density as the temperature increased.^100^

#### 2.3.2. Viscosity

Viscosity characterizes how a fluid responds to deformation under a specific shear rate. Understanding the viscosity behaviour of any liquid is crucial because viscosity measurement provides insights into the molecular-level interactions within the liquid phase. The viscosity of DESs has been the subject of extensive research due to its significant relevance in industrial settings. It is a key factor in assessing their suitability as a medium for chemical reactions. Considerable discrepancies in the viscosity of DESs arise from differences in viscosity measurement methods, various DES preparation techniques, and the potential presence of impurities in the samples.^101^ Furthermore, there are noticeable variations in the viscosity of commonly used DESs when prepared through heating and grinding.^91^

The viscosity of DESs is primarily determined by the chemical properties of the substances involved, which are known as hydrogen bond acceptors (HBAs) and hydrogen bond donors (HBDs). For example, when ChCl is mixed with ethylene glycol in a 1 : 4 ratio, the resulting DES has a low viscosity of 19 cP at 293.15 K. However, if the HBD is replaced with ZnCl_2_, the viscosity of the DES significantly increases to 8500 cP at 298.15 K.^102^ Similarly, DESs that include carbohydrates such as xylitol and sorbitol or carboxylic acids such as malonic acid tend to have much higher viscosities. For example, a ChCl/sorbitol DES can have a viscosity of 12 730 cP at 293.15 K and a ChCl/malonic acid DES can have a viscosity of 1124 cP at 298.15 K.^103,104^ This increase in viscosity is due to the formation of a network of intermolecular hydrogen bonds. It is also important to note that the molecular structure of the HBD, including its size and weight, can significantly affect how the DES flows. In some cases, such as ChCl : ethylene glycol, ChCl : glycerol and ChCl : urea DESs, the “choline cation” moves more slowly than the HBD. This can be observed when comparing the movement of ChCl with malonic acid, where malonic acid forms longer molecular chains, resulting in slower motion.

The molar ratio of hydrogen bond acceptor (HBA) to hydrogen bond donor (HBD) is a significant factor affecting the viscosity of DESs. In general, when the molar ratio of HBA to HBD is higher, DESs tend to exhibit increased viscosity. This phenomenon can be attributed to the reduction in free volume within DESs, given that they adopt a more compact structure. This decrease in available free volume results in slower molecular motion, and consequently higher DES viscosity.^105^ The introduction of choline chloride (ChCl) in glycerol disrupts the hydrogen bonding, leading to a reduction in the viscosity of the DES.^106^ This trend is similarly observed in DESs such as ChCl : 1,4-butanediol and TBABr : PEG.^102,107^ The water content in deep eutectic solvents (DESs) is crucial for understanding their viscosity. DESs have a strong affinity for water, and thus it is essential to account for their water content. For instance, in a choline chloride (ChCl) : urea (1 : 2) DES, the viscosity decreased from 527.3 cP to 200.6 cP with just 0.1 mol fraction of water.^100^ The presence of water can significantly reduce the viscosity of DESs by factors ranging from 10 to 30, depending on the specific hydrogen bond donors (HBDs) used. In the case of the ChCl : oxalic acid (1 : 1) DES, its viscosity dropped by approximately 44 cP when it absorbed 19.40% moisture. Thus, it is important to note that the viscosity of a DES can be controlled by adding a known amount of water.^91^

Temperature also impacts the viscosity of DESs. Generally, as the temperature increases, the viscosity of DESs decreases. This means that DESs with a high viscosity at room temperature can be used at higher temperatures. For example, the viscosity of ChCl : urea (1 : 2), ChCl : glycerol (1 : 2), ChCl : malonic acid and ChCl : glucose decreased from 750 to 95 mPa s, 259 to 52 mPa s, 1124 to 161 mPa s, and 7992 mPa s to 262 mPa s in the temperature range of 298 K to 328 K.^102,104^ The temperature-dependent viscosity of DESs can be explained using the hole theory, a concept explored by researchers including Abbott and colleagues.^48^ According to this theory, the viscosity and electrical conductivity of DESs depend on the presence of “holes” within the liquid, which facilitate the movement of ionic compounds within the network. Instead of strong interactions between hydrogen bond acceptors (HBA) and hydrogen bond donors (HBD), this theory emphasizes that the viscosity is mainly influenced by volume-related factors. The hole theory also helps in understanding the impact of steric effects and interactions on the viscosity of DESs.^108^

These “holes” or empty spaces have been studied in choline and tetrabutylammonium bromide-based DESs containing substances such as phenol, glycerol, ethylene glycol and malonic acid.^61^ The distribution of these holes varies depending on the types of HBA and HBD. The hole theory suggests that when ionic materials melt, they create tiny empty spaces due to the change in liquid density with temperature. These small holes are in constant motion. At lower temperatures, they are very small compared to the components of DESs, making it difficult for the components to fit. This restricts their mobility and results in higher viscosity, typically in the range of 100 to 1000 Pa at lower temperatures. As the temperature increased, the average hole size became more compatible with the size of the DES components. This allowed smaller components to move more freely into these holes, enhancing the overall mobility of the DES. The hole theory assumes that these cavities within the DES move in the opposite direction to the solvent molecules. Thus, at a given temperature, a component of the DES can only move if there is a hole available that matches its size.

Researchers often use two models to study how temperature affects the viscosity of deep eutectic solvents (DESs), *i.e.*, the Arrhenius model and the Vogel–Fulcher–Tammann (VFT) model.^50,100,109^ The Arrhenius model connects the viscosity of DESs with temperature using parameters such as pre-exponential constants and activation energy (*E*_a_). It reveals that less viscous DESs, such as ChCl : ethylene glycol, have lower *E*_a_ values, while highly viscous DESs such as ChCl : glucose have higher *E*_a_ values. For instance, ChCl DES combined with oxalic acid, malonic acid and glutaric acid show *E*_a_ values of −65.2, −46.7, and −47.6 kJ mol^−1^, respectively. Larger *E*_a_ values indicate strong hydrogen bonding, which significantly influences the viscosity of DESs. The VFT model offers an alternative way to describe the connection between the viscosity and temperature of DESs. It uses parameters such as *A*, *B*, and *T*_0_, where *T*_0_ represents the Vogel temperature.^100,110^ Researchers have also developed empirical models, such as the Eyring-based viscosity model, to establish links between temperature and composition-dependent viscosity.^111^ The choice among these models depends on the specific DES composition.

### 2.4 Applications of DESs

#### 2.4.1. Extraction of natural products

**Fig. 1:**
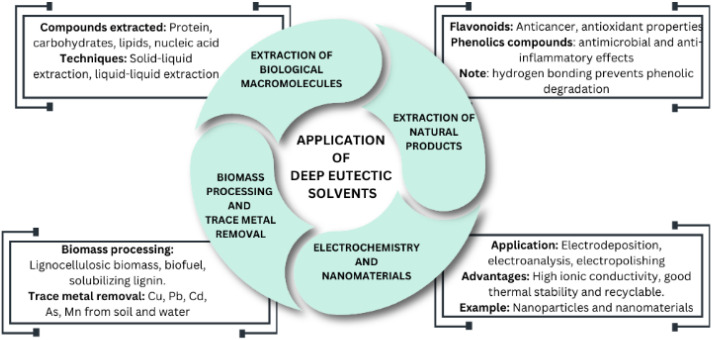
Application of DESs in various fields, including natural product extraction, macromolecule extraction, electrochemistry, nanomaterials, biomass processing, and trace metal removal.

Fig. 1 shows the application of DESs in various fields, including natural product extraction, macromolecule extraction, electrochemistry, nanomaterials, biomass processing, and trace metal removal. Natural products found in nature encompass a variety of compounds, including flavonoids, phenols, polysaccharides, lignins, alkaloids and volatile oils. Extensive research has unveiled their diverse pharmacological effects, ranging from antiviral, anticancer and anti-aging properties. These natural products have found applications in fields such as drug research and new drug development.^112^ For example, flavonoids are valued for their medicinal properties, including antioxidation, anticancer, anti-tumor, anti-allergy and liver protection effects, making their extraction a prominent research focus. Phenolic compounds, another class of natural products, exhibit pharmacological benefits such as antioxidant, antibacterial, anti-inflammatory and antidiabetic properties.^112,113^

The hydrogen bonding between DESs and phenolic compounds prevents the degradation of the phenolic molecules by reducing their movement, thus decreasing their contact time with air, and simultaneously avoiding oxidative degradation.^114^ DESs can be designed for the highly efficient extraction of specific compounds, improving their bioactivity and stability compared to traditional solvents.^115^ Importantly, DESs/NADESs have demonstrated high efficiency in extracting phenolic compounds and offer an eco-friendly alternative to toxic organic solvents. The success of the extraction process is influenced by factors such as solvent composition, component structure, molar ratio, extraction temperature, solid–liquid ratio and water content in DESs/NADESs.

#### 2.4.2. Extraction of biological macromolecules

DESs have contributed significantly to the development of sustainable extraction processes for a wide array of bioactive compounds. Nevertheless, it is worth noting that the application of DESs in the extraction of biological macromolecules remains relatively limited to date. Biomacromolecules, which serve as fundamental building blocks of life, can be found in abundance within the natural world from sources such as plants, animals, and microorganisms.^116,117^ These biomacromolecules, encompassing proteins, carbohydrates, lipids and nucleic acids, have attracted significant interest due to their unique structural and functional characteristics, making them valuable components in a variety of industries including the pharmaceutical, biomedical, cosmetics and food industries.^118^

The uses of DESs as extraction solvents for proteins falls into two main categories, *i.e.*, solid–liquid extraction and liquid–liquid extraction. In solid–liquid extraction, analytes are dissolved in a DES, which serves as the liquid solvent, originating from solid samples. Alternatively, liquid–liquid extraction involves the partitioning of compounds of interest into one of two immiscible phases, with one of these phases being the DES.^119,120^ In a separate study, Saravana and team applied a combination of subcritical water and DES (ChCl : glycerol) to extract polysaccharides from brown seaweed, specifically *Saccharina japonica*.^121^ Researchers developed three hydrophobic deep eutectic solvents by combining oleic acid with terpenes such as menthol, geraniol and thymol. These novel solvents were designed for extracting astaxanthin, a carotenoid pigment, from the microalgae *Haematococcus pluvialis*.^122^

#### 2.4.3. DESs in electrochemistry and preparation of nanomaterials

The electrical conductivity, broad potential windows and excellent metal ion solubility of DESs make them suitable for various electrochemical applications, including electrodeposition, electropolishing, electroanalysis, metal extraction and refining. Thus, an opportunity exists to create cost-effective liquid solutions that can rival traditional electrochemical electrolytes.^123^ This potential lies in using concentrated metal salt hydrates, especially those rich in calcium and magnesium, which can also be more environmentally friendly. DESs offer advantages as well, known for their safety, cost-effectiveness and recyclability when used as electrolytes in electrochemical reactions.^124^

DESs have gained significant attention for their versatile properties, including impressive thermal stability, effective dispersion capabilities, substantial ionic conductivity and wide electrochemical window. These attributes have led to their utilization in various applications, such as serving as dispersants, exfoliants and templates for nanomaterials. Their role in facilitating the synthesis of nanoparticles, both through chemical and electrochemical methods, is similar to the use of ionic liquids in these contexts.^112^ The ongoing progress in nanotechnology with the help of DESs is likely to be highly valuable for advancing various fields such as medicine, energy storage, catalysis and carbon capture. This is achieved through the creation of new and innovative nanomaterials.^125,126^

#### 2.4.4. DESs in biomass processing and determination of trace metals

Biomass processing involves the use of plant waste to produce biofuels and biochemicals through fermentation processes.^127,128^ However, much of the plant mass, including lignin and biopolymers, remains unused. The challenge is pre-treating the raw materials, with existing methods being energy intensive, environmentally harmful, and expensive.^129–131^ Accordingly, DESs are promising solvents for addressing these issues, given that they are capable of solubilizing, extracting and producing value-added products from lignocellulosic materials.^132–134^ For example, Kumar *et al.* demonstrated the solubility of rice straw lignin in NADES as a pretreatment option, achieving 60% solubility and extraction purity exceeding 90%.^135^

The existence of trace metals in soil poses substantial risks to both food safety and the environment. These trace metals can have natural origins or result from the excessive application of metal-based fertilizers, pesticides and other human activities. Consequently, the evaluation of trace metal concentrations in the soil plays a vital role in ensuring environmental safety and advancing agricultural practices. DESs have demonstrated their effectiveness in extracting heavy metals such as Cu, Pb, Cd, As and Mn, from various sources, including food water, and soil, achieving removal rates exceeding 90%.^136–138^ Traditional analysis techniques, including dry/wet digestion, ultrasonic-assisted extraction, microwave-assisted acid digestion and similar methods, often involve the use of dangerous chemicals such as sulfuric acid, hydrochloric acid, nitric acid and oxidants with halogen ions.^136–138^ Thus, to address the environmental and health risks associated with these hazardous substances, there is an urgent requirement to develop eco-friendly reagent preparation methods.

### 2.5 Critical perspectives on DES development and applications

Deep eutectic solvents (DESs) have emerged as a groundbreaking innovation in green chemistry, offering a versatile and environmentally friendly alternative to conventional solvents. Their unique characteristics, such as low toxicity, affordability, biodegradability, and ease of preparation, have positioned them at the forefront of sustainable extraction technologies. However, despite these advantages, the full potential of DESs has yet to be realized. The broad range of compositions and classifications of DESs, while enabling tailored solutions for specific applications, presents challenges in standardization and reproducibility. For example, the overlapping properties of various DES categories, such as natural DESs (NADES) and therapeutic DESs (THEDES), often blur the boundaries of their classification, complicating their systematic study. Moreover, the physicochemical properties of DESs, including high viscosity and sensitivity to water content, may limit their performance in certain industrial processes, necessitating further optimization.

From a practical perspective, the hydrogen-bond-based formation mechanism of DESs underscores their adaptability, but the reliance on these interactions also makes them susceptible to variations in external conditions, such as temperature and humidity. These characteristics highlight both the strength and limitations of DESs, particularly compared to ionic liquids, which share similar attributes but often lack the biodegradability and cost-efficiency of DESs. Moving forward, research efforts should focus on addressing these challenges by exploring novel DES compositions, improving their scalability for industrial use, and enhancing their compatibility with diverse chemical and biological systems. By bridging these gaps, DESs can unlock new opportunities in sustainable extraction processes, particularly for bioactive compounds from natural sources, and set a new standard for green chemistry solutions.

## 3. DESs for extraction of bioactive compounds from plant

**Fig. 2:**
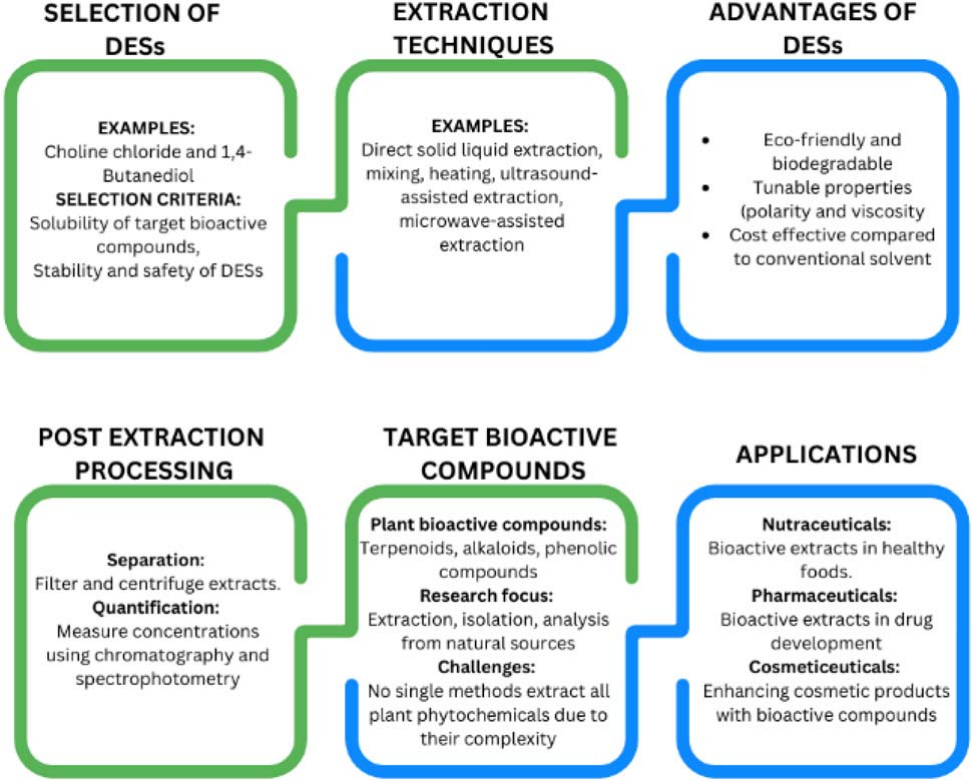
Important elements of DESs for the extraction of bioactive compounds from plants.

Fig. 2 shows the important elements of DESs for the extraction of bioactive compounds from plants. The growing focus on healthy nutrition has driven the rapid expansion of the global market for functional foods and dietary supplements. Simultaneously, there is an increasing interest in substituting synthetic additives and colorants with natural alternatives. This shift extends beyond the food industry, with similar demands in the pharmaceutical and cosmetic sectors. Bioactive compounds in plants, such as terpenoids, alkaloids, sulfur-containing compounds, nitrogen-containing compounds and phenolic compounds, have significant effects on humans and animals.^139,140^ Phenolic compounds, a specific group of bioactive compounds, are gaining recognition for their value in enhancing functional food production and replacing synthetic additives due to their numerous health benefits. As a result, numerous studies have been conducted to refine the extraction, isolation, separation and analysis of these compounds from natural sources. However, due to the diverse and complex structures of phenolic compounds, there is no universally applicable method for extracting all the subclasses of plant polyphenols.^141^Table 1 provides insights into the application of DESs for the extraction of bioactive compounds from plants in selected research.

**Table 1:** Extraction of phytochemicals from the selected plants using DESs with their specific target compounds

Plant/biological sample	Type of DES	Target compound	Comments	Citation	Reference number
*Pyrola incarnata* Fisch.	DES-MAE	Phenolic compounds	Choline chloride : 1,4-butanediol (1 : 4), 30% water	Yao *et al.*, 2015	142
*Acanthopanax senticosus*	DES-UAE	Phytochemicals	Choline chloride : lactic acid (1 : 2), 20% water (v/v)	Shi *et al.*, 2020	143
*Herba Epimedii*	DES-UAE	Flavonoids	l-Proline–ethylene glycol (1 : 4)	Guo *et al.*, 2020	144
*Oroxylum indicum* Vent.	DES-TSE	Flavonoids	Choline chloride : 1,4-butanediol (1 : 3), 40% water	Yin *et al.*, 2020	145
*Prunella vulgaris* L.	DES	Phenolic acids	Choline chloride : ethylene glycol (1 : 4), 30–36% vol water	Xia *et al.*, 2015	146
*Chamaecyparis obtusa*	HS-SDME	Bioactive terpenoids	Choline chloride : ethylene glycol (1 : 4)	Tang *et al.*, 2014	147
Traditional Chinese medicines	DES-HF-LPME	Cinnamic acid derivatives	Chloride : hexanoic acid (1 : 3)	Zhang *etal.*, 2020	148
Olive leaves	DES-HSE	Phenolic compound	Choline chloride based : acetic acid (1 : 2), 50% water addition	de Almeida Pontes *et al.*, 2021	149
*Ampelopsis grossedentata*	DES-UAE	Flavanoids	Choline chloride : glucose (4 : 1), 20% water	Zhen *etal.*, 2022	150
Mate tea	DES-UAE	Phenolic compound	Lactic acid : glycine : water (3 : 1 : 3)	Rebocho *et al.*, 2022	151
			Lactic acid : glucose : water (5 : 1 : 3)		
*Moringa oleifera* L.	DES-UAE	Phenolic compound	Proline : glycerol (2 : 5), 37% water content	Wu *et al.*, 2020	152
*Eucommia ulmoides*	DES-MAE	Phenolic acids (chlorogenic acid)	ChCl–1,4-butanediol–ascorbic acid (1 : 1 : 0.2)	Yu *etal.*, 2021	153

The extraction and separation of bioactive compounds from plants using deep eutectic solvents (DES) primarily involve direct solid–liquid extraction supported by techniques such as mixing, heating, ultrasound and microwaving. Following this extraction, further separation of the compounds is typically carried out through chromatography, with a focus on high-performance liquid chromatography (HPLC) equipped with either ultraviolet (UV) or mass spectrometric (MS) detectors. Several studies have explored DES-based extraction followed by the quantification of bioactive compounds in herbal samples. In a study in 2015, the polyol-based DES of choline chloride : 1,4-butanediol combined with microwave-assisted extraction was employed to extract five phenolic compounds from *Pyrola incarnata* Fisch., identifying the optimal DES with specific conditions that enhanced the extraction efficiency compared to heat-stirring and ultrasonic extraction.^142^ Another study conducted in 2020 employed a DES of choline chloride : lactic acid for the ultrasonic-assisted extraction (UAE) of compounds from *Acanthopanax senticosus* roots, which enabled the extraction of a wide range of phytochemicals, making DESs potential replacements for strong organic solvents in certain situations.^143^ An efficient DES-UAE procedure using l-proline–ethylene glycol (1 : 4) for evaluating the quality of *Herba Epimedii*, a herbal mixture, was reported in 2020, selecting a particular DES with reduced solvent use and extraction time, showcasing its advantages over traditional methods.^146^

A tissue-smashing extraction (TSE) procedure was introduced for extracting flavonoids from seeds, demonstrating an effective DES that yielded higher extraction efficiency and shorter extraction time compared to methanol.^145^ DES has found application in various microextraction techniques. A DES-based procedure for extracting rosmarinic acid and salviaflaside from solid *Prunella vulgaris* L. samples was developed utilizing a specific alcohol-based DES (choline chloride–ethylene glycol at a 1 : 4 M ratio).^146^ This method, employing ultra-performance liquid chromatography (UPLC) for quantification, outperformed traditional techniques such as ultrasonic and maceration. An effective headspace single-drop microextraction (HS-SME) method was developed for extracting bioactive compounds, showing a superior performance compared to other extraction techniques.^147^ Another study investigated the application of deep eutectic solvent-based hollow fibre liquid-phase microextraction (DES-based HF-LPME) for analyzing compounds in Chinese medicines, providing valuable insights into the extraction and determination mechanisms.^148^ The extraction of phenolic compounds from olive leaves was assessed, comparing the efficiency of a specific deep eutectic solvent (DES) composed of choline chloride and acetic acid to ethanol. The results emphasized the superior performance of the ChCl : acetic acid DES when exposed to specific temperature and water addition conditions, resulting in a 15% higher extraction of phenolic compounds from olive leaves compared to ethanol.^149^

Ultrasound-assisted extraction with a natural deep eutectic solvent (NADES) also has been employed for efficient flavonoid extraction from *Ampelopsis grossedentata* leaves. The most suitable NADES consisted of choline chloride and glucose (4 : 1 molar ratio, 20% water), resulting in an 83.93% flavonoid yield and significant bioactivities. This approach makes it a promising option for plant-derived functional foods.^151^ In a different study, they used a new way to extract antioxidants from mate tea leaves, even the decaffeinated ones, using natural deep eutectic systems (NADES). This method produced two separate extracts, *i.e.*, one with pigments and the other with polyphenols, and it was more effective than traditional methods, extracting 30% more polyphenols. Also, the antioxidants remained stable for at least three months when placed in NADES, and the absence of caffeine did not affect this.^151^ A study improved the extraction of antioxidant-rich compounds from *Moringa oleifera* L. leaves using DES-based ultrasonic extraction under specific conditions. These conditions included a water content of 37%, power of 144 W and temperature of 40 °C, which outperformed other methods. HPLC analysis found 14 phenolic compounds, including high levels of vicenin-2 and orientin.^152^

## 4. Therapeutic potential of bioactive compounds extracted from mango

Mango (*Mangifera indica* L.) belongs to the Anacardiaceae family and has a history of cultivation in Asia for over 4000 years.^154^ The key mango varieties dominating the global export market include Tommy Atkins, Haden, Ataulfo, Kent, Keitt, and Alphonso.^155–158^ In Southeast Asia, various mango varieties are cultivated, such as ‘Chokanan’, ‘Golden Phoenix’, ‘Water Lily’, ‘Harumanis’, ‘Sala’, ‘Masmuda’, and others.^159–163^ Mangoes are popular for their bioactive compounds such as polyphenols, carotenoids, and vitamins. Mango bark has been traditionally used to treat diarrhea, cancer, diabetes, and skin infections, and as a diuretic and antiseptic.^164^ With a history of centuries of medicinal uses, mangoes exhibit significant promise in offering a range of therapeutic benefits, such as antioxidative, anti-inflammatory, anti-cancer and anti-diabetic effects. Globally, a significant challenge arises from the generation of substantial agricultural and food waste due to industrial processes and consumer consumption, leading to environmental contamination. Mango by-products stand out for their abundant bioactive compounds, including mangiferin, phenolic acids, flavonoids and tannins. These compounds provide antioxidant properties that can combat diseases such as cancer and cardiovascular issues. Moreover, these by-products exhibit antimicrobial effectiveness against a range of harmful microorganisms. Table 2 provides insights into the potential health benefits, usage of diverse mango components, and mango varieties that have been examined in selected research. Mangoes are a great source of antioxidants such as carotenoids, vitamin C (ascorbic acid) and different types of phenolic compounds.^165,166^ These phenolic compounds include flavonoids, phenolic acids, xanthones, and gallotannins.^167,168^ It is important to note that the composition of these compounds can differ depending on the type of mango. Two specific compounds of interest are ascorbic acid (vitamin C) and mangiferin. Ascorbic acid plays a key role in protecting against oxidative damage caused by reactive oxygen species (ROS) and has neuroprotective properties. Mangiferin, found in mangoes, is most concentrated in their fruit peel and stem bark.^168,169^ It helps prevent oxidative reactions and can neutralize various types of ROS, similar to vitamin C.

**Table 2:** Therapeutic potential of mango varieties or cultivars and their sample parts

Therapeutic potential	Example of variety/cultivar and sample parts used	Citation	Reference number
Antioxidant properties	Tommy Atkins (peel, seed, and leaves), Kent (peel, seed, and leaves), Keitt (peel, seed, and leaves), and Haden (peel, seed, and leaves), and Ataulfo mango (seed and peel)	Manthey *et al.*, 2009	165, 170 and 171
Anti-inflammatory	Mahajanaka mango, Keitt mango, Willard mango (peel, pulp and seed kernel), Vellai Colomban mango (peel, pulp and seed kernel), and Karutha Colomban mango (peel, pulp and seed kernel)	Kim *et al.*, 2018; Kim *et al.*, 2017, Kuganesan *et al.*, 2017	172–175
Anti-cancer properties	Hagar mango (peel and seed kernel), Francis mango, Kent mango, Ataulfo mango, Tommy Atkins mango, Haden mango and Okrong mango (leaves)	Shaban *et al.*, 2023; Noratto *et al.*, 2010; Ganogpichayagrai *et al.*, 2017	176–178
Anti-diabetic properties	Variety information is not clear (stem bark and flowers), Anwar Ratol mango (leaves) and Okrong mango (leaves)	Akram *et al.*, 2014; Mistry *et al.*, 2023; Saleem *et al.*, 2019; Ganogpichayagrai *et al.*, 2017	164 and 178–180

Inflammation is a natural and important immune system response to injuries, infections, or stress, serving as a fundamental part of the healing and defence mechanisms in the body. There are instances where inflammation can persist for extended periods and lead to various health problems. “Anti-inflammatory” involves methods or substances aimed at mitigating or preventing excessive inflammation within the body. Mango extracts and compounds such as gallic acid and mangiferin were studied for their anti-inflammatory effects in mice with colitis. This study showed that mango polyphenols improved intestinal health, reduced pro-inflammatory substances, and eased colitis symptoms by influencing specific pathways.^172,173^ Gallic acid blocks inflammation by binding to the IGF-1R receptor.^181^ Mangiferin reduces inflammation in mouse colitis models through various mechanisms.^182^ In another study, mango leaf extracts from three different varieties (Morakot, Nam Doc Mai, and Mahajanaka) were examined to explore their potential in reducing inflammation and acting as antioxidants.^174^ To assess inflammation, nitric oxide release was measured in a lab setting, and various antioxidant tests were performed. The MeOH extract from the Mahajanaka variety (M-MeOH) demonstrated the most significant effect and was further evaluated for its ability to reduce inflammation in rats, where it showed noteworthy results. Additionally, the presence of mangiferin, a beneficial compound found in mango leaves, was confirmed in both the MeOH and aqueous extract.

The formation of tumors disrupts normal cell growth and reduces cell death. Recent research highlights a significant link between tumor development and the inhibition of a crucial process called apoptosis.^183^ Apoptosis is typically identified by specific morphological features and energy-dependent biochemical processes. Apoptosis is a regulated cellular mechanism that typically occurs in various natural situations, such as growth, development, reproduction and the maintenance of healthy organs and tissues.^176^ Mango extracts combated DMBA-induced breast cancer through distinct methods. These extracts served as potent antioxidants, reducing oxidative stress and restoring the antioxidant balance. They also displayed anti-proliferative and pro-apoptotic effects by increasing caspase-3 activity, promoting DNA fragmentation and elevating caspase-3 expression.^176^ Each extract offered therapeutic benefits against DMBA-induced mammary tumors, supporting apoptosis and countering oxidative stress, proliferation and estrogenic effects. Their role in pharmacology is significant.^177^ Research has been conducted to explore the potential of polyphenolic extracts from different mango varieties, including Francis, Kent, Ataulfo, Tommy Atkins, and Haden, in inhibiting cancer cell growth.^176,177^ Extracts from Ataulfo and Haden, selected for their strong antioxidant properties, delivered the most promising results. Specifically, Ataulfo, at a concentration of 5 mg GAE per L, significantly suppressed the growth of SW-480 colon carcinoma cells by 72%, without affecting noncancer colonic myofibroblast CCD-18Co cells. This growth inhibition in SW-480 cells was associated with increased pro-apoptotic markers, cell cycle regulation, cell cycle arrest and a reduction in the production of reactive oxygen species.^177^

Diabetic animals show significant elevations in blood sugar, cholesterol and triglycerides, accompanied by decreased body weight and glycogen reserves. The quest for effective diabetes treatments is a worldwide concern. Researchers are investigating non-pharmaceutical methods to complement or substitute traditional treatments. Furthermore, in traditional medicine, different parts of *M. indica* are employed for managing diabetes. Mango peel extract and mangiferin improved the condition of rats with alloxan-induced diabetes.^179^ These rats initially lost weight but began to gain it back after the second week, which the control group did not experience. After 21 days of treatment, their high blood sugar levels significantly decreased. The treatments also increased the glycogen levels in muscles and the liver, aiding in diabetes management. Additionally, the extracts had useful effects on the cholesterol and triglyceride levels and helped restore pancreatic function in diabetic rats.^179^ In a different study, it was found that an extract from *M. indica* cv. Anwar Ratol leaves contained helpful compounds such as mangiferin.^180^ This extract showed promise for managing diabetes by lowering blood sugar after meals, improving glucose tolerance, increasing body weight, improving lipid profiles and reducing harm to beta cells. Thus, the use of this leaf extract from the plant can be beneficial for diabetes management and related issues such as weight loss and lipid profile changes. Research has been conducted to explore the potential of *M. indica* cv. Okrong, specifically its leaf extract and mangiferin, in contributing to the management of diabetes and potentially combating cancer.^178^ They were tested for their ability to inhibit enzymes related to diabetes, and both showed dose-dependent inhibition. Additionally, the mango leaf extract displayed potential in killing cancer cells at higher concentrations. This suggests that *M. indica* cv. Okrong can be beneficial for diabetes management and potentially for fighting cancer.^178^

## 5. Phytochemical extraction from different varieties and parts of mango by DES

The information regarding the selected optimum DESs used for the extraction of phytochemicals from different mango varieties and the specific parts employed for extraction is presented in Table 3. The study conducted by Pal and Jadeja in 2020 focused on the extraction of valuable compounds from ripe Kesar mango peels.^37^ The researchers used a unique mixture of lactic acid, sodium acetate and water as the extracting solution, enhancing the process with the use of a microwave. Through a systematic approach, this team determined the most effective extraction conditions, which included using a microwave with a power of 436.45 W, an extraction time of 19.66 min, and a specific amount of liquid for every gram of peel. Under these optimal conditions, a significant quantity of valuable compounds was successfully extracted, with mangiferin identified as the predominant compound.^37^ The combination of microwave assistance and the specialized liquid demonstrated remarkable efficiency in the extraction process. Additionally, when these extracts were added to sunflower oil, they significantly extended its shelf life. This discovery underscores the potential of mango peels, which are often discarded, as a valuable source of antioxidants when utilizing this specific solution and the microwave technique.

**Table 3:** Selected optimum DESs used for the extraction of phytochemicals from different mango varieties and the specific parts employed for extraction

Sample	DES	Extraction procedure	Compound extract	Operating conditions	Extraction yield/conversion efficiency	Findings	Citation & reference number
Mango peel (Kesar mango)	Lactic acid : sodium acetate : water (3 : 1 : 4)	HSE and MAE	Phenolic compounds	HSE, magnetic stirrer: 500 rpm, temperature: 60 °C, time: 90 min, liquid-to-solid ratio: 50 : 1 (mL g^−1^)	HSE > 80 mg GAE per g DW	- HPLC analysis discovered mangiferin as the prominent phenolic compound in the mango peel extracts	Pal and Jadeja, 2020 (ref. 37)
					MAE 56.17 mg GAE per g DW	- Lactic acid : sodium acetate DES system in combination with MAE showed remarkable effects on the extraction efficiency of phenolic compounds	
				MAE, power of 436.45 W, time of 19.66 min, liquid-to-solid ratio: 59.82 mL g^−1^			
Mango peel (Jinhuang mango)	Betaine : citric acid (3 : 1), 30% water (before optimization)	HSE and UAE	Pectin	Bet : CA pH: 0.85, liquid–solid ratio of 27 : 1, temperature: 80 °C, water content of Bet–CA: 70%	Bet–CA 27.63%	- Two eco-friendly solvents extracted higher pectin yields than conventional hydrochloric acid	Chen *et al.*, 2022 (ref. 38)
	Choline chloride : malic acid (1 : 2), 30% water (before optimization)						
				ChCl : MaA pH: 0.40, temperature: 85 °C, time: 120 min, water content of ChCl–MaA: 70%, liquid–solid ratio: 27 : 1	ChCl : MaA 30.01%	- High intensity ultrasound treatment remarkably increased the extraction yield of low-ester pectins, but decreased their molecular weight and particles size	
Mango peel (Alphonso mango)	Lactic acid : glucose (5 : 1), 20% water	UAE	Phenolic compounds	Water content: 20%, duty cycle: 50%, acoustic density: 2 W cm^−3^, liquid : solid ratio 30 : 1 (v/w), particle size: 0.3 mm, time: 30 min	69.85 mg GAE per g	- UAE in combination with NADES technique offered 1.4 times TPC, 1.7 times TFC, 1.9 times antioxidant activity	Lanjekar *et al.*, 2022 (ref. 39)
						- UA-NADES also offered 50% shorter time, and 25% reduced solvent consumption compared to the batch method with 80% ethanol	
Mango leaves (Gedong mango)	Lactic acid : sodium acetate (3 : 1) in distilled water	MAE	Alkaloids, flavonoids, saponins and tannins	Temperature: 70 °C, time: 1 h	Qualitative test of the secondary metabolites	- Screening of phytochemical compounds found that the extract of Gedong mango leaves contains polyphenol	Rizikiyan *et al.*, 2022 (ref. 184)
						- The use of Gedong mango and mulberry extract in spray gel preparation revealed the sunscreen activity	
Mango peel	Glycerol : sodium acetate (3 : 1), with 20% water	MAE	Phenolic compounds	MAE, power: 440.18 W, time: 12.10 min, liquid to solid ratio: 59.99 mL g^−1^	MAE 155.28 mg GAE per g DW	-The extraction rate was very slow for Gly : SA DES system (without the combination of MAE methods) due to their higher viscosity	Pal and Jadeja, 2022 (ref. 185)
						-Higher than other organic solvents like distilled water or 70% (v/v) methanol	

**Fig. 3:**
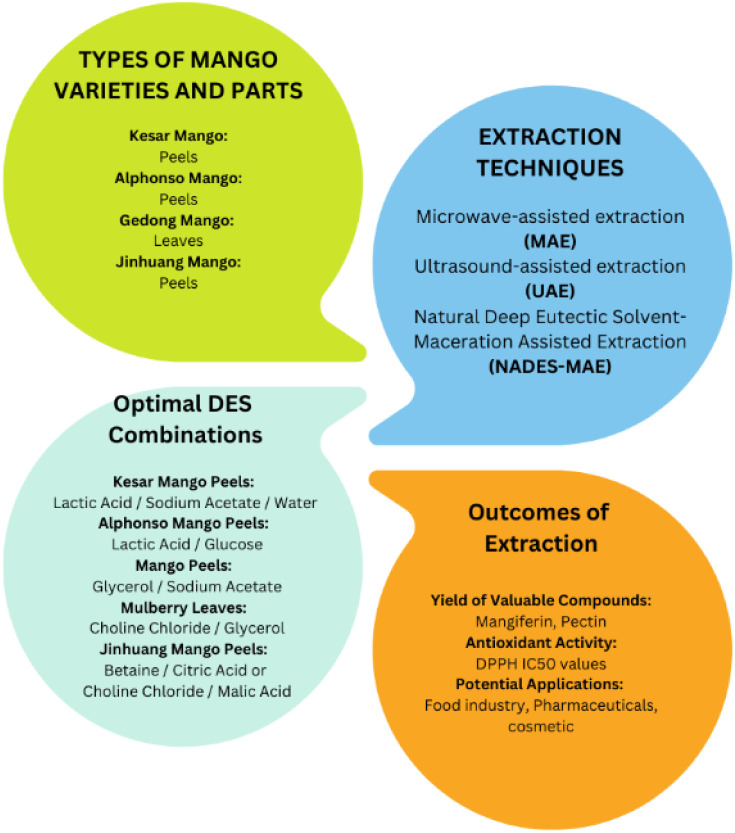
Phytochemical extraction from different varieties and parts of mango using DESs.

Fig. 3 shows the extraction of phytochemicals from different varieties and parts of mango using deep eutectic solvents (DES). In 2022, the same research team explored the use of microwave-assisted extraction to convert mango peels into valuable polyphenolic compounds.^185^ This innovative process not only utilized clean energy but also aimed to repurpose discarded peels for bioenergy production, including biofuels, heat and electricity. Mango peels, rich in potent antioxidant mangiferin, are often overlooked despite the environmental concerns related to their disposal. The researchers used a DES and created a technique called microwave-assisted deep eutectic solvent extraction (MADESE). Among the different DES options, glycerol : sodium acetate proved to be the most effective for extracting the polyphenolic content from mango peels. They employed advanced techniques to optimize the process, achieving the best conditions with 155.28 mg of polyphenolic content per gram of dry weight, outperforming other solvents.^185^ Although the glycerol : sodium acetate DES had a slower extraction rate due to its higher viscosity, MADESE holds significant potential for the large-scale extraction of bioactive compounds, particularly in the food and medical industries.

Lanjekar and colleagues highlighted the potential of Alphonso mango peels as a valuable source of compounds, with a specific focus on polyphenol extraction.^39^ Their research effectively addressed the limitations associated with traditional organic solvents by introducing natural deep eutectic solvents (NADESs) as an eco-friendlier and more non-toxic alternative. The extraction process was optimized by combining NADES with ultrasound, leading to impressive results. Using an NADES mixture containing lactic acid and glucose in a 5 : 1 ratio, this study achieved the highest total phenolic content (TPC) at 69.85 mg GAE per g of mango peel, a total flavonoid content (TFC) of 16.5 mg QE per g of mango peel, and a DPPH IC_50_ value of 35.37 μg mL^−1^, indicating its potent antioxidant activity.^39^ The ultrasound-assisted NADES method significantly improved the efficiency by providing higher TPC and TFC levels, increased antioxidant activity, shorter extraction time, and reduced solvent consumption compared to traditional ethanol-based extraction. The characterization and quantification of the polyphenolic profile revealed that gallic acid was the predominant antioxidant, followed by chlorogenic acid, with quercetin and kaempferol as the major flavonoids.

Rizikiyan *et al.* (2022) initiated a research project with the primary goal of formulating an effective sunscreen spray gel.^184^ To achieve this objective, they employed the natural deep eutectic solvent-maceration-assisted extraction (NADES-MAE) method, a recognized technique for extracting bioactive compounds from plant materials. This investigation focused on the utilization of Gedong mango and mulberry leaves, with specific types of deep eutectic solvents (DES) meticulously prepared for this purpose. For the extraction of Gedong mango, an NADES solvent consisting of sodium acetate and lactic acid in a 1 : 3 ratio with distilled water was used, and the MAE extraction was carried out for one hour at 70 °C. Simultaneously, mulberry leaves were subjected to extraction using a DES prepared with choline chloride and glycerol in a 1 : 2 ratio, with the extraction process conducted for approximately one hour at 80 °C.^184^ Following extraction, the resulting extracts were subjected meticulous filtration and evaporation processes to eliminate any remaining solvents. The findings of this study strongly suggest that the combination of these extracts through the NADES method, using DES, holds significant promise for application in sunscreen formulations, particularly in the form of a spray gel. This research represents an exciting step forward in the development of natural, plant-based sunscreen products.

Mango peels from the Jinhuang variety have been reported to be used for the extraction of pectin with DESs.^38^ In this investigation, two novel, environmentally friendly DESs including betaine : citric acid and choline chloride : malic acid were explored for pectin extraction from mango peels. The extraction conditions were optimized, and ultrasound significantly influenced the extraction yield and pectin properties. The pectins extracted with these DESs exhibited higher yields, larger sizes and increased particle dimensions compared to hydrochloric acid (HCl) extraction. The use of high-intensity ultrasound further increased the yield of low-ester pectins, while reducing their molecular size and particle dimensions. The analysis revealed a higher galacturonic acid (GalA) content and more extensive homogalacturonan (HG) regions in the pectins obtained with the DESs.^38^ These findings offer insights into potential applications in the food and pharmaceutical industries, providing a better understanding of the connection between extraction conditions and the physical and chemical characteristics of mango peel pectins when using DESs.

**Fig. 4:**
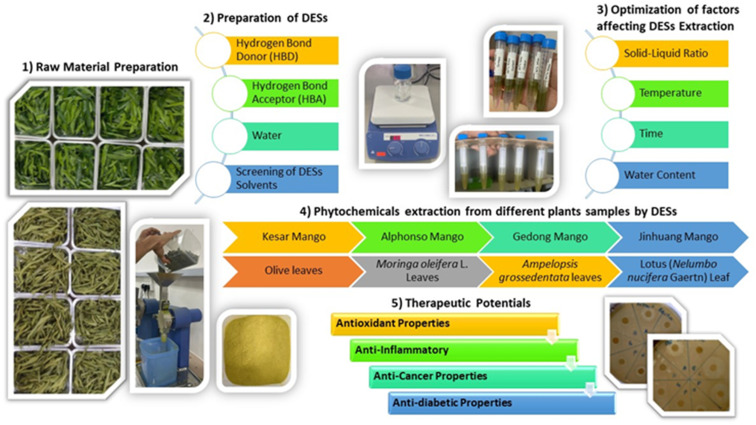
Basic aspects of green extraction using DESs, with various mango varieties and their therapeutic potential.

Besides the optimal DESs, the phytochemicals from mango have also been screened with different sets of DESs. These DESs were tested with varying ratios and applied to specific parts of the mango (Table 4). For instance, choline chloride and urea in a 1 : 2 ratio were used to extract phytochemicals from mango peel, particularly from Kesar mango.^37^ Similarly, choline chloride and sorbitol in a 3 : 1 ratio were applied to mango peel from the same variety. Various other DES combinations, such as choline chloride with different organic acids, were employed for the extraction of mango peel from Jinhuang mango.^38^ Sodium acetate, in combination with glycerol and lactic acid, was used for extraction from Kesar mango peel and Gedong mango leaves.^37,184^ These studies aimed to optimize the use of DESs for the extraction of phytochemicals from mango samples, with a focus on different mango varieties and plant parts. Fig. 4 depicts the fundamental aspects of extracting bioactive compounds using DESs, various mango varieties, and their therapeutic potential. The manuscript review suggests that the predominant practice in mango DES extraction focuses on employing peels, while the exploration of mango leaves and kernels for this purpose was relatively limited. The exploration towards the use of leaves is valuable given that it opens up the potential for a more comprehensive understanding of the bioactive components in the mango plant and their diverse applications. In the next section, we review the extraction of phytochemicals from leaves of other plants, further highlighting the significance of investigating alternative sources for phytochemical extraction.

**Table 4:** Different DES (deep eutectic solvents) ratios experimented and applied to distinct parts of mango samples

Component 1	Component 2	Tested ratio	Extraction parts	Variety	Citation & reference number
Choline chloride	Urea	1 : 2	Mango peel	Kesar mango	Pal *et al.*, 2020 (ref. 37)
Choline chloride	Sorbitol	3 : 1	Mango peel	Kesar mango	Pal *et al.*, 2020 (ref. 37)
Choline chloride	Sucrose	1 : 1	Mango peel	Kesar mango	Pal *et al.*, 2020 (ref. 37)
Choline chloride	Glycerol	1 : 3	Mango peel	Kesar mango	Pal *et al.*, 2020 (ref. 37)
Choline chloride	Glycerol	—	Mango peel	Alphonso mango	Lanjekar *et al.*, 2022
Choline chloride	Lactic acid	1 : 3	Mango peel	Kesar mango	Pal *et al.*, 2020 (ref. 37)
Choline chloride	Lactic acid	—	Mango peel	Alphonso mango	Lanjekar *et al.*, 2022 (ref. 39)
Choline chloride	Malic acid	1.5 : 1	Mango peel	Jinhuang mango	Chen *et al.*, 2022 (ref. 38)
Choline chloride	Glucose	—	Mango peel	Alphonso mango	Lanjekar *et al.*, 2022 (ref. 39)
Choline chloride	Malic acid	—	Mango peel	Alphonso mango	Lanjekar *et al.*, 2022 (ref. 39)
Choline chloride	Malic acid	1 : 2	Mango peel	Jinhuang mango	Chen *et al.*, 2022 (ref. 38)
Choline chloride	Citric acid	2 : 1	Mango peel	Jinhuang mango	Chen *et al.*, 2022 (ref. 38)
Choline chloride	Oxalic acid	1 : 1	Mango peel	Jinhuang mango	Chen *et al.*, 2022 (ref. 38)
Choline chloride	Levulinic acid	1 : 2	Mango peel	Jinhuang mango	Chen *et al.*, 2022 (ref. 38)
Choline chloride	Acetic acid	1 : 3	Mango peel	Jinhuang mango	Chen *et al.*, 2022 (ref. 38)
Sodium acetate	Glycerol	1 : 3	Mango peel	Kesar mango	Pal *et al.*, 2020 (ref. 37)
Sodium acetate	Glycerol	1 : 3	Mango peel	Kesar mango	Pal *et al.*, 2022 (ref. 185)
Sodium acetate	Lactic acid	1 : 1, 1 : 2, 1 : 3, 1 : 4	Mango peel	Kesar mango	Pal *et al.*, 2020 (ref. 37)
Sodium acetate	Lactic acid	1 : 3	Mango leaves	Gedong Mango	Rizkiyan *et al.*, 2022 (ref. 184)
Betaine	Citric acid	1 : 2	Mango peel	Jinhuang mango	Chen *et al.*, 2022 (ref. 38)
Betaine	Oxalic acid	1 : 2	Mango peel	Jinhuang mango	Chen *et al.*, 2022 (ref. 38)
Betaine	Levulinic acid	1 : 1	Mango peel	Jinhuang mango	Chen *et al.*, 2022 (ref. 38)
Betaine	Malic acid	1 : 2	Mango peel	Jinhuang mango	Chen *et al.*, 2022 (ref. 38)
Betaine	Acetic acid	1 : 3	Mango peel	Jinhuang mango	Chen *et al.*, 2022 (ref. 38)
l-Proline	Citric acid	1 : 2	Mango peel	Jinhuang mango	Chen *et al.*, 2022 (ref. 38)
l-Proline	Oxalic acid	1 : 2	Mango peel	Jinhuang mango	Chen *et al.*, 2022 (ref. 38)
l-Proline	Levulinic acid	1 : 2	Mango peel	Jinhuang mango	Chen *et al.*, 2022 (ref. 38)
l-Proline	Malic acid	1 : 2	Mango peel	Jinhuang mango	Chen *et al.*, 2022 (ref. 38)
Lactic acid	Glucose	5 : 1	Mango peel	Alphonso mango	Lanjekar *et al.*, 2022 (ref. 39)

## 6. Phytochemical extraction from leaves of different plants by DES

Table 5 presents examples of deep eutectic solvents (DESs) used for extracting compounds from leaves, other than mango samples. DESs were employed to extract phytochemicals from olive leaves, with a specific focus on the combined use of choline chloride and carboxylic acid for the extraction of phenolic compounds.^149^ This study compared the efficiency of this method to ethanol-based extraction and utilized response surface methodology to investigate the influence of temperature and the addition of water on both deep eutectic solvent (DES) and ethanol extraction processes. The study aimed to address the existing knowledge gap concerning the behaviour of choline chloride and acetic acid when used in combination. Furthermore, it characterized their solid–liquid equilibrium (SLE), density and viscosity, both with and without the addition of water. When choline chloride and acetic acid were employed at a temperature of 54.1 °C, with 50.0% water addition, there was a significant 15% increase in phenolic compounds compared to ethanol extraction at the same temperature with only 0.5% water added.^149^ Additionally, the SLE analysis revealed the formation of a eutectic region at a molar ratio of approximately 1 : 2 for choline chloride and acetic acid.

**Table 5:** Examples of DESs used for extracting compounds from the leaves of plants other than mango samples

Sample	DES selected for bioactive compound extraction	Extraction procedure	Compound extract	Operating conditions	Extraction yield/conversion efficiency	Findings	Reference
Olive leaves	Choline chloride based : acetic acid (1 : 2), 50% water addition	HSE	Phenolic compound	Temperature: 54.1 °C, addition of water: 50.0%	470.03 mg kg^−1^	- Choline chloride–acetic acid yielded 15% more phenolic compounds	de Almeida Pontes *et al.*, 2021 (ref. 149)
						- The new class of solvents extracted two times more phenolic compounds than ethanol	
*Moringa oleifera* L. leaves	Proline : glycerol (2 : 5), 37% water content	UAE	Phenolic compound	Water content: 37% ultrasonic power: 144 W, ultrasonic temperature: 40 °C	45.9 mg GAE per g DW	- Comparative study confirmed that the optimized DES-based UAE yielded further higher TPC, TFC and antioxidant activities than other extraction methods	Wu *et al.*, 2020 (ref. 152)
Olive leaves	Glycerol : lysine (3 : 1), 10% water concentration	Batch extraction procedure (HSE)	Phenolic compounds	Liquid to solid ratio: 150 mL g^−1^, water concentration (v/v): 10%, temperature: 80 °C	188.39 mg per g GAE DM	- Glycerol–lysine DES was found to be the most effective in extracting phenolic compounds from olive leaf compared with glycerol–arginine DES and glycerol–proline and even to conventional solvents such as ethanol, methanol and water	Akli *et al.*, 2022 (ref. 186)
*Mitragyna speciosa* Korth. Havil leaves	Choline chloride : sorbitol (3 : 1)	MAE	Polyphenol	Liquid–solid ratio: 20 mL g^−1^, time: 20 min, microwave power (% of watts): 60%	539.37 μg GAE per g	- The combination of NADES and MAE with some different combinations of NADES composition is more effective than a maceration	Prabowo *et al.*, 2021 (ref. 187)
						- SEM imaging result shows that the levels of damage of cells and cell walls were more severe after extraction	
*Ampelopsis grossedentata* leaves	Choline chloride : glucose (4 : 1), 20% water	UAE	Flavanoid	Liquid-to-solid ratio: 30 mL g^−1^, ultrasonication power: 490 W, ultrasonication time: 6.5 min	83.93%	- The actual flavonoid yield was 83.93%, which was close to the predicted yield	Zhen *et al.*, 2022 (ref. 150)
						- Further, 86.75% of the flavonoids were recovered by adding the same volume of phosphate buffer saline (100 mM, pH of 7.0) to the extract solution	
Walnut leaves (*Juglans regia* L.)	Choline chloride : butyric acid (1 : 2), 20% water (before optimization)	HAE	Phenolic compound	Time: 180 min, temperature: 30 °C, water proportions: 53%	37.9 mg per g DW	- The water content was clearly the most relevant extraction variable, followed by temperature and, lastly, extraction time	Vieira *et al.*, 2018 (ref. 188)
Mandarin leaves	Citric acid : glycerol (1 : 4) 50% water	MAE	Phenolic and flavonoid ingredients	Time: 90 s, temperature: 80–90, 30 °C, microwave power: 500 W	14.47 mg GAE per g DS	- Citric acid–glycerol DES is one of the solvents that has shown the best performance for bioactive compound extraction	Kurtulbaş *et al.*, 2019 (ref. 189)
Mate tea leaves	Lactic acid : glucose : water (5 : 1 : 3)	UAE	Phenolic compound	Time: 60 min, solid/liquid ratio: 1 : 20 (w/v, g mL^−1^), temperature: 40 °C	Lactic acid : glucose : water: 12.2 mg GAE/100 mg mate tea leaves	- Lactic acid-based system showed to be more efficient in extracting phenolics	Rebocho *et al.*, 2022 (ref. 151)
	Lactic acid : glycine : water (3 : 1 : 3)				Lactic acid : glycine : water: 13.5 mg GAE/100 mg mate tea leaves	- NADES systems were able to extract 30% more of polyphenolic components of the mate tea leaves matrices, when compared with traditional solvents/techniques	
Lotus (*Nelumbo nucifera* Gaertn.) leaf	Lactic acid : glycerol (1 : 2), 20% of water before optimization	HAE	Phenolic and flavanoid	Water content: 29%, liquid–solid ratio: 37 : 1 mL g^−1^, extraction time: 61 min, extraction temperature: 53 °C	Flavonoids: 126.10 mg g^−1^	- The extraction of lotus leaves using the NDES method was superior to both ethanol extraction and water extraction	Yang *et al.*, 2023 (ref. 190)
					Polyphenols: 126.10 mg g^−1^		
*Eucommia ulmoides* leaves	ChCl–1,4-butanediol–ascorbic acid (1 : 1 : 0.2)	MAE	Phenolic acids (chlorogenic acid)	Temperature: 53.03 °C, time: 20.08 min, solid–liquid ratio: 18.53 mL g^−1^, power: 500 W	CGA = 3.659 mg g^−1^	Integrated microwave-assisted ternary deep eutectic solvent extraction, which was first applied to extract compounds from EUL for the first time	Yu *et al.*, 2021 (ref. 153)

Deep eutectic solvents (DESs) have also been utilized for the extraction of phytochemicals from olive leaves using different sets of chemicals. This innovative type of liquid was formulated by blending glycerol with lysine, proline and arginine.^186^ These DESs were tested for the very first time to determine their effectiveness in extracting healthful compounds from olive leaves. To optimize the extraction process, they used a specially designed plan to find the right amount of liquid, the strength of the DES and the temperature. The ideal conditions involved using 150 mL of liquid for every gram of olive leaves, with the DES at 90% concentration, and maintaining a temperature of 80 °C.^186^ These conditions delivered the expected results, yielding approximately 188.39, 100.01, and 95.97 mg of healthful compounds for different DES systems. The top performer was the glycerol–lysine DES, outperforming conventional solvents including ethanol, methanol and water. In fact, it extracted 40–71% more total phenol yield compared to the regular solvents. Further testing showed that the glycerol–lysine DES was particularly effective at extracting specific healthful compounds.

Wu *et al.* employed a UAE method with a specific deep DES to enhance the extraction of total phenolic and flavonoid content (TPC/TFC) and antioxidant properties from *Moringa oleifera* L. leaves.^152^ They optimized the process using the response surface methodology (RSM) and central composite design (CCD). The results showed that the water content in the DES had a significant impact on all the responses, while ultrasonic time and the liquid-to-solid ratio did not affect the total phenolic content. The optimal conditions for achieving the best TPC/TFC and antioxidant activities included 37% water content in DES, 144 W ultrasonic power and a constant ultrasonic temperature of 40 °C, consistent with the predicted results. This optimized DES-based UAE method yielded higher TPC, TFC and antioxidant activities compared to other extraction techniques, with the HPLC analysis confirming the presence of 14 phenolic compounds, including substantial concentrations of vicenin-2 (17.6 mg g^−1^) and orientin (23.6 mg g^−1^) in the extracts from *M. oleifera* leaves.^152^

The DESs were used to extract polyphenols from *Mitragyna speciosa* (Korth.) Havil leaves through the microwave-assisted natural deep eutectic solvent extraction (MANDESE) method.^187^ This method was optimized using the response surface methodology (RSM). The extraction process was examined through scanning electron microscopy (SEM) imaging. Two extraction methods, maceration and MANDESE, were applied, and the determination of total polyphenol content was carried out using Folin–Ciocalteu reagent and a UV-vis spectrophotometer. SEM imaging was employed to assess any cell and cell wall damage. The optimization factors considered in RSM included the composition ratio of the natural deep eutectic solvent (NADES), the liquid–solid ratio, extraction time and microwave power. The results highlighted the superior efficiency of MANDESE with various NADES compositions compared to maceration. SEM imaging revealed an increased level of cell and cell wall damage following extraction. The optimal conditions included an NADES composition ratio of 3 g g^−1^ (choline chloride/sorbitol), a liquid–solid ratio of 20 mL g^−1^, a 20 min extraction time and 60% microwave power. A scale-up test confirmed a total polyphenol content of 526.12 μg GAE per g sample.^187^

Zhen *et al.* conducted an ultrasound-assisted extraction with natural deep eutectic solvents (NADESs) for the efficient extraction of flavonoids from *Ampelopsis grossedentata* leaves.^150^ They identified the optimal NADES composition of a 4 : 1 molar ratio of choline chloride to glucose with 20% water. The extraction process was further fine-tuned with a liquid-to-solid ratio of 30 mL g^−1^, ultrasonication at 490 W for 6.5 min, resulting in 83.93% flavonoid yield, closely aligning with their prediction.^150^ By adding an equivalent volume of phosphate buffer saline to the extract, they successfully recovered 86.75% of the flavonoids. Although the flavonoid extract displayed slightly reduced chemical antioxidant activity compared to dihydromyricetin, it effectively inhibited the proliferation of human breast MDA-MB-231 cells. This inhibition was achieved by inducing apoptosis, altering the cell cycle, modulating the mitochondrial membrane potential, and mitigating intracellular reactive oxygen species (ROS).

The extraction of phenolic compounds from walnut leaves (*Juglans regia* L.) involved optimizing the process through heat-assisted extraction and deep eutectic solvents based on choline chloride and carboxylic acids.^188^ Preliminary screening of various carboxylic acids as hydrogen bond donors identified the most effective mixture of choline chloride with either butyric or phenylpropionic acid at a 1 : 2 mole ratio, together with 20% water (w/w). Subsequently, the extraction conditions, including time, temperature and water proportion, were fine-tuned using the experimental design and response surface methodology. This study quantified the three most abundant compounds (neochlorogenic acid, quercetin 3-*O*-glucoside and quercetin *O*-pentoside) identified by HPLC. Additionally, they explored the effect of the solid/liquid ratio under the optimal conditions, finding no significant decrease in extraction efficiency until a concentration of 140 g L^−1^.^188^

DESs, in conjunction with microwave-assisted extraction (MAEX), have been employed for the extraction of valuable antioxidative phenolic and flavonoid compounds from Satsuma mandarin leaves.^189^ This innovative approach involves using deep eutectic solvents (DESs), which are a blend of citric acid and glycerol as hydrogen bond acceptor (HBA), and glycerol, dimethyl urea, and methylimidazole as hydrogen bond donors (HBD). The Satsuma mandarin leaves were first dried and ground before undergoing extraction using these DESs, together with conventional solvents such as water, ethanol, methanol, ethyl acetate, acetonitrile and their water solutions. Subsequently, the extracted samples were evaluated for their total phenolic content (TPM) and total flavonoid content (TFM) through UV-spectrophotometry, while their antioxidant capacities were assessed by measuring their inhibition activity towards DPPH.^189^

Rebocho *et al.* presented an innovative method for the selective extraction of antioxidants from both regular and decaffeinated mate tea leaves using a variety of NADES.^151^ Their study incorporated a systematic approach, optimizing the extraction parameters such as the solid-to-liquid ratio, temperature, extraction time, stirring and the integration of ultrasound-assisted extraction (UAE) technology. Their research findings demonstrated that by employing a two-step extraction process, initially utilizing a hydrophobic system (Men : Lau, 2 : 1) followed by a hydrophilic lactic acid-based NADES, two distinct extracts were obtained, one rich in pigments and the other abundant in polyphenols. In comparison to conventional solvents and techniques, the NADES systems exhibited a remarkable 30% increase in the extraction of polyphenolic components from the mate tea leaves matrices.^151^ Furthermore, the incorporation of the extract into the NADES medium, rather than into an aqueous solution, significantly enhanced the antioxidant stability, maintaining its functionality for a minimum of three months and resulting in an impressive 41% improvement compared to the extracts obtained through traditional methods. Importantly, the absence of caffeine in the extracts did not appear to impact the stability results.

Yang *et al.* employed NADES to extract flavonoids from lotus leaves and optimized the process using the Box–Behnken design method.^190^ The best conditions involved using NADES comprised of lactic acid and propanetriol at a 1 : 2 molar ratio with a 29% water content, a solid–liquid ratio of 37 : 1 (mL g^−1^), and extraction at 53 °C for 61 min, resulting in a flavonoid yield of 126.0972 mg g^−1^ and a polyphenol yield of 113.1163 mg g^−1^, aligning with the model prediction. This method not only increased the flavonoid yield but also enhanced the antioxidant activity compared to traditional extraction methods. LC-MS analysis of the NADES extract revealed the extraction of 19 compounds, highlighting its efficiency.^190^

DESs were harnessed for an environmentally friendly extraction process aimed at obtaining iridoids and phenolic acids from *Eucommia ulmoides* Oliver leaves (EUL).^153^ This innovative approach, named integrated microwave-assisted ternary deep eutectic solvent extraction (TDES-MAE), marked its debut in EUL compound extraction. Within this specialized TDES setup, the addition of a small quantity of ascorbic acid (Vc) adjusted the acidity of binary deep eutectic solvents (BDES), formed from a 1 : 2 M ratio of choline chloride and 1,4-butanediol, to create TDES. This adapted solution showed a superior extraction yield. Several factors, including DES type, molar ratio, water content and choice of hydrogen bond donor (HBD), were explored to identify the most effective TDES solvent. Using the Box–Behnken design, the optimal conditions such as extraction time, extraction temperature and liquid–solid ratio were determined through the response surface methodology (RSM). Under these ideal conditions, the TDES-MAE method boosted the extraction efficiency by a significant 1.4-fold compared to traditional methods.^153^

## 7. Important factors affecting phytochemical extraction by DES

### 7.1 Effect of water and viscosity

**Fig. 5:**
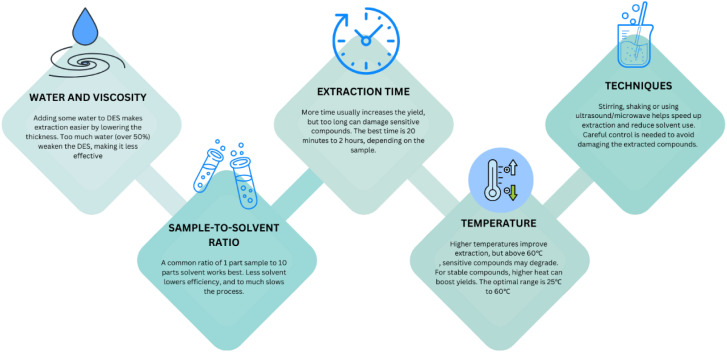
Important factors that affect phytochemical extraction *via* deep eutectic solvents (DES).

The introduction of water into DESs can induce significant alterations in their physiochemical properties and the integrity of their hydrogen bonding network, as reported by Vilková *et al.* in 2020.^191^ This approach enables the customization of the DES characteristics to align with the specific requisites of various processes. However, the inclusion of water can produce two distinct effects, *i.e.*, it reduces the viscosity, thus enhancing the extraction efficiency, decreasing waste production and contributing to cost-effectiveness. Conversely, exceeding the optimal water content weakens the hydrogen bond network within the DES and its interactions with analytes, potentially resulting in diminished extraction efficiency.^192–194^Fig. 5 shows the important factors that affect phytochemical extraction using deep eutectic solvents (DESs).

Precise control of the water content in deep eutectic solvents (DESs) is crucial for their use as extraction solvents, given that an excessive water content, exceeding 50%, can disrupt the hydrogen bond framework and influence the viscosity of DESs.^44^ High viscosity is a common challenge when using DESs or NADESs as extraction solvents, limiting their ability to penetrate the extraction matrix. Although increasing the temperature during extraction can reduce the viscosity, it is not always the best choice due to the energy consumption and potential presence of heat-sensitive phytochemicals. Thus, to address challenges such as high viscosity and the associated costs of using pure DESs as the extraction medium, researchers have practically employed aqueous NADES solutions, which typically consist of a water concentration in the range of 20% to 80%. Existing literature suggests that an optimal equilibrium is achieved at a water content of approximately 20% to 30%.^195,196^ This equilibrium allows the maintenance of a fluid extraction system while preserving the essential hydrogen bond structure of the DES. Nevertheless, the potential of a higher water content to disrupt this structure should not overshadow their suitability for specific extraction scenarios. Precise control of the water content in DES systems has emerged as a critical consideration when optimizing DES-based extraction processes.

For example, Koutsoukos *et al.* used water as a co-solvent to extract phenolic compounds from brown propolis with ChCl/tartaric acid NADES, and they used methanol as a co-solvent for extracting carotenoids from apricot pulp and shrimp head by-products using the same NADES.^197^ The concentration of water in the DES-water or NaDES-water mixtures significantly influenced the efficiency of extraction. Bi *et al.* determined that a ChCl/1,4-butanediol NaDES mixture with 35% water is the optimal medium for extracting myricetin and amentoflavone from *Chamaecyparis obtusa*.^198^ Similarly, Zhao *et al.* found that efficient extraction of rutin from *Sophora japonica* flower buds can be achieved using ChCl/triethylene glycol DES containing 20% water.^199^ Their studies on the viscosity of 20 DESs showed that their viscosity increased with the formation of more hydrogen bonds formed among their constituents.

### 7.2 Effect of sample-to-solvent ratio (SLR)

The sample-to-solvent ratio (SLR) is a crucial factor in deep eutectic solvent (DES)-based extraction processes, and its optimal balance is vital to ensure both efficiency and scalability. The SLR dictates the proportion of solid sample immersed in the solvent, making it a key determinant of extraction performance. When only a small sample amount is introduced into the solvent, the extraction process may prove inefficient, especially when being scaled up to handle larger quantities of material. The limited sample processing in this case can hinder the overall extraction efficiency, making it less effective and time-consuming when dealing with larger volumes of material in industry.

Alternatively, using an excess of solid sample compared to the solvent presents its own set of challenges. The surplus solid material can impede the efficient dispersion of the solvent, resulting in slower interactions between the sample and the solvent. Consequently, this may reduce the contact surface area between the sample and the solvent, potentially diminishing the overall performance of the system. To strike the right balance, many studies have undertaken extensive investigations, often through statistical analyses or referencing prior successful works. They have typically converged on a solid SLR ratio of 1 : 10, which is considered an effective compromise.^200,201^ This ratio allows the processing of a sufficient quantity of sample, while maintaining the efficiency of the extraction method. Researchers and practitioners widely embrace this ratio as a practical and reliable approach for DES-based extraction processes, given that it ensures both efficient extraction and scalability, meeting the needs of various applications and settings.

### 7.3 Effect of time

The extraction time is a crucial parameter in the extraction of phytochemicals such as phenolic compounds using DES. It significantly influences the efficiency and yield of the extraction process by determining the duration during which the phenolic compounds are in contact with the DES. Generally, longer extraction times lead to higher yields, given that they allow more extensive interactions between the phenolic compounds and the solvent. However, it is important to consider the kinetics of the extraction process, given that there is usually an initial rapid increase in yield, which may slow down over time as the system approaches equilibrium. Therefore, the choice of extraction time should consider the specific kinetics of the extraction system. The optimal extraction time may vary depending on factors such as the type of phenolic compounds, the specific plant material and the type of DES used. Achieving this optimal time requires experimentation and optimization to fine-tune the extraction duration for the specific application.

It is also essential to consider the potential impact of prolonged exposure to DES and heat on the stability of thermolabile phenolic compounds, given that excessive heat may lead to degradation. Most of the processes reviewed have very high retrieval percentages, with extraction times ranging from 20 min to 2 h.^37,39,196^ Naturally, the type of extraction also defines the extraction time required, with energy-assisted methods such as heating, ultrasound or microwave requiring less extraction time, but more energy to conduct. Overall, the use of DESs has enabled undeniably short extraction times for all extraction methods employed. When scaling up the extraction process, careful consideration of the required extraction time is necessary to maintain efficiency and consistency. In summary, the extraction time significantly affects both the quantity and quality of the extracted phenolic compounds, and finding the right balance is essential for effective and efficient DES-based extractions.

### 7.4 Effect of temperature

The temperature at which extraction processes are conducted significantly influences the duration, efficiency and overall performance of the extraction. Generally, higher temperature enhances the mobility of molecules, expediting the diffusion of extracted substances into the solvent. This principle remains valid when utilizing deep eutectic solvents (DESs). However, it is important to note that DES-based extractions are not exempt from the impact of temperature, given that they depend on temperature to mitigate the high viscosities inherent to DESs, which can hinder the smooth operation of the extraction process. According to the literature, the optimal temperature for extraction typically falls in the range of room temperature (around 25 °C) to approximately 60 °C.

For example, the temperature used for the extraction of phytochemicals using DESs was 30 °C, 40 °C and 60 °C for walnut leaves, *Moringa oleifera* L. leaves and Kesar mango peel, respectively.^37,152,188^ However, it is worth considering that excessively high temperatures have their downsides. They not only consume more energy, which deviates from the environmentally friendly “green” aspect of extraction, but also pose potential risks to both the integrity of the DES and the stability of the target substances, especially given the thermal sensitivity of many natural compounds involved.^202^ Furthermore, extremely high temperatures have been found to decrease the extraction yields in certain cases. This reduction in yield is attributed to the decline in the interaction between the target compound and the chosen solvent, irrespective of their individual thermal resistance. It is essential to underscore that the thermal characteristics of the substances used remain a limiting factor in these instances.^195^

### 7.5 Effect of auxiliary

During the extraction process, various techniques are employed to mix samples, which include stirring, vortexing, shaking and combinations of heating and stirring. Each of these approaches has an array of advantages and disadvantages. These methods are considered auxiliary, given that they provide additional support to the extraction procedure. For example, considering a sample of medicinal plant material subjected to ultrasound-assisted extraction (UAE), UAE can significantly reduce the extraction time for bioactive compounds, making it a valuable technique in the pharmaceutical and herbal medicine industries. By utilizing ultrasound, the extraction process becomes more efficient, enabling the extraction of bioactive compounds such as flavonoids, alkaloids and essential oils from medicinal plants. This method is not only faster but also helps preserve the integrity of these valuable compounds.

On the other hand, microwave radiation offers several benefits, including expedited extraction, reduced usage of solvents and reagents, precise control of heating and efficient energy transfer. For instance, when extracting essential oils from aromatic herbs, microwave-assisted extraction (MAE) has been employed to enhance the extraction efficiency. The combination of microwave radiation with deep eutectic solvents (DESs) results in a swift and efficient extraction process, yielding high-quality essential oils that find applications in the perfume and cosmetics industries.^203^ Additionally, MAE reduces the environmental impact by minimizing the solvent usage. For example, Jinhuang and Alphonso mangoes have been subjected to DES extraction combined with UAE, while Kesar and Gedong mangoes have been exposed to DES with MAE.^37–39,184^ However, both ultrasound and microwave radiation can induce alterations in the structure of the target analytes, potentially leading to their degradation or modification, even when DESs are used as the extraction medium. Consequently, it is imperative to meticulously fine-tune the extraction conditions to attain the desired outcomes, while mitigating any adverse effects.^203^

## 8. Assessment of bioactive compounds extracted *via* DESs

For a thorough analysis of bioactive compounds, the process begins with careful sample preparation. This involves drying the samples using controlled temperature and humidity or freeze-drying, followed by turning them into a consistent powder through sieving. Various factors such as pH, moisture, protein, ash, fiber, acidity, lipids, and sugars are assessed. Subsequently, DESs are used to extract these compounds, and techniques such as HPLC and GC are employed to identify and measure them. Thus, ensuring that DESs can maintain the stability of these compounds in the extract is crucial, considering factors such as heat, light, storage time and environmental conditions, especially sunlight, to ensure their reliability for various uses. Additionally, other tests are often performed to evaluate how DESs affect the properties of the extract, including determining total phenolic content (TPC), total flavonoid content (TFC), antioxidant activity and antimicrobial activity.^204^

Phenolic compounds are characterized by hydroxyl groups linked to an aromatic ring. These compounds are classified into simple phenols, polyphenols, coumarins and other categories.^63^ Researchers are increasingly interested in phenolic compounds because of their noteworthy and beneficial properties. The total phenolic content is commonly assessed through spectrophotometric means, utilizing a specific redox reagent known as the Folin–Ciocalteu reagent. This method enables the determination of the total concentration of phenolic hydroxyl groups in plant extracts, with the results typically expressed as gallic acid equivalent (in milligrams of gallic acid per gram of dried sample).^205,206^

Flavonoids, a subgroup of phenolic compounds with a carbon-based core structure, have gained attention due to their antioxidant, antimicrobial and antitumor properties, especially in the context of bladder cancer. Traditional methods for extracting flavonoids rely on various techniques and conventional solvents, primarily because these compounds are challenging to dissolve in aqueous solutions, leading to time and material loss. Recently, DESs have emerged as a more efficient and cost-effective alternative for flavonoid extraction, yielding higher quantities. The determination of total flavonoid content (TFC) is typically carried out using the aluminium chloride colorimetric method, which is based on the ability of aluminium to form stable complexes with flavones, flavonols, and specific dihydroxyl groups found in flavonoids. Various reference standards, such as quercetin, catechin and rutin, are used for TFC measurement.^206–208^

Various methods for evaluating antioxidant activity, such as the DPPH free radical scavenging assay, ferric reducing antioxidant power (FRAP), hydroxyl radical scavenging activity and ABTS, have been utilized to assess the effectiveness of different extraction approaches. For example, the extraction of phenolic and flavonoid compounds from marjoram using a deep eutectic solvent (DES) composed of lactic acid, glycine and water in a 3 : 1 : 3 molar ratio resulted in higher yields compared to a 60% aqueous ethanol solvent. This extract exhibited substantial antiradical activity, measuring 1950 μmol DPPH per gram of dry weight.^209^ Similarly, in the case of *Rosmarinus officinalis* L., the use of a DES mixture of choline chloride and lactic acid in a 1 : 3 ratio resulted in the extraction of higher levels of phenolic compounds compared to the traditional 100% ethanol as the solvent. Subsequent analysis of the phenolic compounds in the extract indicated a remarkable ferric reducing antioxidant property, with a value of 183.82 mM Trolox per gram, surpassing that obtained using an ethanol extract.^210^

Researchers have explored the cytotoxic effects of medicinal plant extracts obtained through deep eutectic solvents (DES) using a MTS assay *in vitro*. Polyphenolic compounds extracted from grape pomace with choline chloride : citric acid (2 : 1) were tested against HeLa (cervical cancer) and MCF-7 (breast cancer) cell lines, showing 37.61% cell viability at 5% concentration (v/v) within 72 h. A similar protocol for extracting compounds from olive pomace also demonstrated significant *in vitro* cytotoxicity (MTS assay) with 12.19% cell viability at the same concentration within the same time frame.^211^ Moreover, ginsenoside, a bioactive compound from ginseng, was extracted using a ternary DES (GPS-5) glycerol : l-proline : sucrose (9 : 4 : 1) as an alternative solvent, revealing anti-tumor activity against human colorectal cancer cell lines at 58 μg mL^−1^, while the DES itself exhibited no cytotoxic effects based on the MTT assay results.^212^

Bacterial growth inhibition testing is a widely employed and efficient method due to its cost-effectiveness and swift results. In one study, phycocyanin, a bioactive compound from *Arthrospira platensis*, was extracted using DESs. Among the tested DES options, a 1 : 1 mixture of xylose and glycerol was proven to be the most effective, exhibiting robust antimicrobial properties against *Escherichia coli* and *Enterobacter aerogenes* with well diameters of 17 mm and 16 mm, respectively. Another investigation assessed the antimicrobial potential of total polyphenols extracted from *Punica granatum* L. using a malic acid : glucose : glycerol (1 : 1 : 1) solvent system. This extract displayed significant inhibitory effects against the Gram-positive bacterium *Staphylococcus aureus*, with a 90% inhibition rate observed at a concentration of 0.7 mg mL^−1^. Importantly, this result differed from the activity of ascorbic acid, suggesting that polyphenols may interact with microorganism cell membranes, potentially leading to microbial cell death or enzyme inhibition.^213^

## 9. Conclusions and future perspectives

Deep eutectic solvents (DESs) represent a transformative advancement in green chemistry, offering an environmentally friendly, cost-effective, and efficient alternative to conventional solvents for the extraction of bioactive compounds. Mango (*Mangifera indica* L.), a globally cultivated fruit with a rich therapeutic profile, has emerged as a promising natural source for these compounds, including polyphenols, carotenoids, vitamins, and minerals. Although mango peels have traditionally been the focus of DES-based extractions, this review emphasized the untapped potential of other parts of the mango plant, such as its leaves, seeds, and kernels, to broaden the spectrum of bioactive compounds.

However, despite their potential, the application of DESs has certain limitations. The high viscosity of certain DES formulations can impede mass transfer, while their sensitivity to water content affects the extraction consistency and efficiency. Additionally, the diverse range of DES compositions and classifications, including natural DESs (NADESs) and therapeutic DESs (THEDES), complicates the standardization and reproducibility in research. These challenges necessitate systematic studies to establish standardized protocols and optimize DES formulations for specific applications.

From a mechanistic perspective, the reliance of DESs on hydrogen-bonding interactions offers both adaptability and vulnerability. External factors, such as temperature and humidity, can alter these interactions, impacting the extraction performance. Future research should explore innovative DES formulations with improved thermal and chemical stability, ensuring their effectiveness across varied operational conditions.

The integration of DESs with advanced extraction techniques, such as microwave-assisted and ultrasound-assisted methods, presents an opportunity to enhance the scalability and reduce the energy demands of processes. These approaches can address the limitations of traditional extraction methods, while maintaining or improving the yield and purity of bioactive compounds. Additionally, leveraging DESs for hydrophobic compound extraction, through hydrophobic DES formulations, can expand their applicability to a broader range of natural products.

The variability in bioactive compound profiles among mango cultivars offers another avenue for exploration. Comparative studies across different mango varieties can uncover unique compounds or higher concentrations of specific bioactives, enabling the development of targeted nutraceuticals and functional foods. This cultivar-focused research can also inform breeding programs aimed at enhancing the health-promoting attributes of mangoes. Furthermore, to move from laboratory-scale studies to real-world applications, rigorous bioavailability and safety evaluations are essential. Preclinical and clinical trials will validate the therapeutic potential of DES-extracted compounds and address concerns related to their stability, absorption, and efficacy in human systems. These efforts will bridge the gap between scientific research and industrial applications, fostering the development of high-value products that cater to health-conscious consumers.

Sustainability is a cornerstone of DES applications. By valorizing agricultural by-products such as mango peels, seeds, and other underutilized components, DES-based extraction aligns with the global goals of reducing waste and minimizing environmental impact. This dual benefit of environmental stewardship and product innovation positions DESs as pivotal technology in green chemistry. Looking ahead, the convergence of DES advancements with industrial needs and sustainability imperatives offers a pathway to revolutionize natural product extraction. Continued interdisciplinary research, encompassing materials science, green chemistry, and bioactive compound studies, will unlock the full potential of DESs, contributing to health, industry, and environmental well-being.

## Data availability

This review article discusses data and information obtained from previously published studies, which are duly cited throughout the manuscript. As such, all data referenced in this work are available within the public domain or can be accessed through the cited sources.

## Author contributions

Ahmad Mukhlis Abdul Rahman: conceptualization and writing. Amirul Ridzuan Abu Bakar: conceptualization and supervision. Ang Qian Yee: resources, writing – review and editing. Mohd Asraf Mohd Zainudin: resources, writing – review and editing. Nik Muhammad Azhar Nik Daud: validation, writing – review and editing. Ahmad Anas Nagoor Gunny: resources, writing – review and editing. Ryan Vitthaya Peron: resources, writing – review and editing. Nurul Husna Khairuddin: resources, writing – review and editing.

## Conflicts of interest

There are no conflicts to declare.
